# Soil Moisture and Prey Life Cycles Promote Seasonal Dietary Shift in a Grassland‐Breeding Migratory Shorebird

**DOI:** 10.1002/ece3.74495

**Published:** 2026-10-11

**Authors:** Renée Veenstra, Yvonne Verkuil, Gerrit Potkamp, Jeroen Onrust, Matty P. Berg, Theunis Piersma, Mohamed Henriques

**Affiliations:** ^1^ BirdEyes, Centre for Global Ecological Change, Faculty of Science & Engineering and Campus Fryslân University of Groningen Leeuwarden the Netherlands; ^2^ Conservation Ecology Group, Groningen Institute for Evolutionary Life Sciences (GELIFES) University of Groningen Groningen the Netherlands; ^3^ Department of Wildlife Cal Poly Humbolt Arcata California USA; ^4^ Section Ecology and Evolution, Amsterdam Institute for Life and Environment Vrije Universiteit Amsterdam Amsterdam the Netherlands; ^5^ Department of Coastal Systems NIOZ Royal Netherlands Institute for Sea Research Texel the Netherlands; ^6^ Science Team Sustainability & Wellbeing, Faculty Campus Fryslân University of Groningen Leeuwarden the Netherlands

**Keywords:** diet, DNA metabarcoding, Lumbricidae, prey selection, Tipulidae

## Abstract

Understanding the dietary flexibility of migratory birds is essential for predicting their resilience to rapid environmental changes. For the continental black‐tailed godwit ( *Limosa limosa limosa* ), precise knowledge of seasonal diets and the underlying causes of prey selection on their breeding grounds has remained limited. This is largely due to challenges of observing foraging in tall vegetation and the rapid digestion of soft‐bodied prey. We utilized DNA metabarcoding of 207 fecal samples to describe the diet of flock‐foraging godwits in southwest Friesland, the Netherlands, during pre‐nesting (March) and post‐nesting (May–June) periods. To assess whether dietary changes were resource or selectivity based, we simultaneously quantified soil macrofauna biomass and measured biophysical variables, including soil moisture content, soil penetration resistance, and vegetation height. Earthworms (Lumbricidae) and leatherjackets (Tipulidae) were the primary prey taxa detected. A significant dietary taxa shift occurred between periods: earthworm relative read abundance (RRA) dropped from 73% pre‐nesting to 21% post‐nesting, while that of leatherjackets increased from 3% to 47%. While earthworms were consumed in proportion to their soil biomass in March, their selectivity declined significantly in May and June as soils became drier and harder. Conversely, leatherjacket consumption was higher in the post‐nesting period as their individual biomass increased. Our results demonstrate that the seasonal shift in godwit diet is caused by an interaction between prey life cycles and habitat conditions that impact prey harvestability. Soil desiccation post‐nesting reduces the detectability and accessibility of earthworms, even when they remain present in the soil, potentially forcing birds to switch to leatherjackets as they become more profitable. As climate change and intensive land use exacerbate soil drying in agricultural grasslands, the window for godwits to access earthworms is narrowing. This study highlights that maintaining high soil moisture is critical not just for prey abundance, but to ensure food remains accessible to meadow birds during the vital fueling period before southbound migration.

## Graphical abstract

In this study, we utilized DNA metabarcoding of 207 fecal samples to describe the diet of flock‐foraging godwits in southwest Friesland, the Netherlands, during pre‐nesting (March) and post‐nesting (May–June) periods. To assess whether dietary changes were resource or selectivity based, we simultaneously quantified soil macrofauna biomass and measured biophysical variables, including soil moisture content, soil penetration resistance, and vegetation height. While earthworms were consumed in proportion to their soil biomass in March, their selectivity declined significantly post‐nesting as soils became drier and harder. Conversely, leatherjacket consumption was higher in the post‐nesting period as their individual biomass increased.

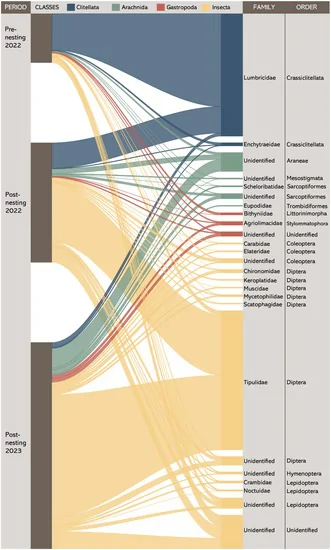

## 1 Introduction

Determining what birds eat, and why, is central to understanding their ecology, behavior, distribution, and population dynamics (Piersma and van Gils 2011). An accurate description of the diet provides the foundational knowledge upon which further ecological studies can be built, allowing, for example, to investigate how populations will respond to local changes in habitat and food resources. Migratory birds are an interesting case in this regard. Being exposed to vastly different habitats along their network of sites, they are known to be fairly sensitive to changes in their environment within and between different moments of their annual life‐cycle (van de Kam et al. 1999).

Many shorebird species, as is the case for birds in general, exhibit seasonal variation in diet composition (Coelho et al. 2024; Gliesch et al. 2023). For example, in the Bijagós Archipelago, Curlew Sandpiper *Calidris ferruginea* eat more polychaetes and less crustaceans over the course of the non‐breeding season, which lasts from October to May. In contrast, Bar‐tailed godwits *Limosa lapponica* start eating more bivalves and less polychaetes toward the end of the season (Coelho et al. 2024). The authors suggest that these patterns may reflect changes in dietary requirements or altered local prey availability. Such changes in food availability may result either from seasonally changing biophysical habitat characteristics (e.g., due to weather conditions) or from prey life cycles that cause temporal shifts in available prey items (Ausden et al. 2003). Investigating dietary change over time can be insightful, as it may provide information on the flexibility of shorebirds to adjust to changes in local food availability (Coelho et al. 2022, 2024).

Prey selectivity is another important aspect potentially linked to seasonal changes in diet composition. Prey items will vary in their nutritional value and on how “easy” they are captured and handled (Zwarts and Wanink 1993). Thus, shorebirds may, for instance, select different prey items at different life cycle moments in response to energy requirements, as is the case when fueling for migration (Martins et al. 2013). Understanding prey selection involves building on an accurate measure of prey availability (Piersma 2012). This requires the detectability and accessibility of different prey to be understood (Zwarts et al. 1992; Zwarts and Blomert 1992), both of which depend on prey behavior and are affected by environmental conditions such as temperature or rainfall. For example, temperature affects the activity of crabs that are key prey for whimbrels *Numenius phaeopus* , which increases their detectability (Zwarts 1990). In such cases, changes in prey selection reflect shifts in prey availability, rather than shifting energy requirements. All these interdependencies can only be explained and further investigated once accurate diet descriptions within different life cycle moments are in place.

Diet descriptions are still lacking for many shorebird species. An example is the continental black‐tailed godwit *Limosa limosa limosa* (hereafter godwit), which spends the non‐breeding period in Western Africa and the Iberian peninsula, and breeds on (agricultural) grasslands in Northwest Europe (Verhoeven et al. 2021). The largest part of the European breeding population of godwits is found in the Netherlands (Kentie et al. 2016), where they arrive in February and March (Lourenço et al. 2011). Whilst recovering from their migration flight, they often forage in flocks on grassland to acquire resources for the upcoming breeding period. Godwits initiate clutches from April onwards, and the peak chick‐rearing period ranges from approximately mid‐May to mid‐June (Kentie et al. 2015). From the end of May, individuals that have finished nesting and raising chicks flock together again and accumulate fuel for the upcoming migration flight southward (Hooijmeijer et al. 2013). Thus far, foraging ecology studies of adult godwits have reported that in grasslands, earthworms Lumbricidae are an important food source (Beintema et al. 1995; Verhoeven et al. 2022). In the period after breeding, leatherjackets, the larvae of craneflies Tipulidae, are consumed instead (Zwarts 1993). However, these studies do not focus on quantifying godwit's diet, and reports of prey species consumed were merely based on visual observations of foraging birds or on soil macrofauna found at nesting sites. Thus, a detailed description of the contribution of different prey items to the godwit diet, which may vary from March to June, is of yet unavailable.

Across the breeding season, the habitat conditions and environmental variables that affect food availability for godwits are likely to shift. For example, as spring progresses and summer approaches, temperatures rise and precipitation generally declines (Twardosz et al. 2021). Such conditions, exacerbated by intensive land‐use practices, may cause desiccation of top soil layers and, consequently, reductions in soil‐dwelling prey populations (Onrust et al. 2019; Onrust and Piersma 2019). Moreover, shifts in insect life cycles may alter the emergence of insect prey from February until June (De Felici et al. 2019). Additionally, drier and harder soils might reduce the accessibility and detectability of subterranean prey by probing godwits (du Toit et al. 2024; Onrust et al. 2019), and sward height may increase the accessibility of above ground prey items such as flying insects (Atkinson et al. 2005). Because of these potential seasonal scenarios it is relevant to assess the diet composition of godwits in relation to soil and vegetation characteristics. This is particularly interesting given that the frequency of extreme climatic events like droughts or floodings has increased over the last decades (Williams and O'Gorman 2022), the consequences of which are amplified by intensified land use, such as maximized grass production in dairy farmland (Kraamwinkel et al. 2021).

In this study, we used DNA metabarcoding analysis of feces to investigate the diet composition of flock‐foraging godwits on grasslands. This molecular method allows us to successfully assess not only the presence of prey species in a bird's diet with high precision (Zhemchuzhnikov et al. 2024), but also to roughly estimate their relative abundance, including those of soft‐bodied species (Verkuil et al. 2022). We determined godwit's diet in the periods before and after nesting, hereafter referred to as the pre‐ and post‐nesting periods. The pre‐nesting period is considered the period after arrival at the breeding grounds and before nesting, whereas the post‐nesting period is considered the period in which nesting and most chick rearing activities have ended, and migratory fueling has started. In these two periods, individuals are not bound to a nest or to escorting chicks and are therefore free to select suitable grassland foraging sites, where food intake is—besides safety (van Gils et al. 2004)—a high priority, making these periods particularly relevant for studying diet composition. Additionally, we assessed whether diets were associated with changes in habitat characteristics under changing (seasonal) weather conditions, food resources, or selectivity for specific prey taxa by godwits.

To achieve our research aim, we (1) described and compared the relative abundance of prey taxa (at the family taxonomic level) in feces of godwits between both periods, (2) identified the most important prey items explaining differences in diet composition between periods, and (3) assessed how the dietary taxa composition was related to biophysical environmental variables potentially impacting prey availability, namely soil moisture content, soil resistance, and vegetation height. Based on the literature (Beintema et al. 1995; Zwarts 1993), we expected that earthworms and leatherjackets would be important prey items for godwits. For those prey items, we (4) measured their fresh biomass in the soil to evaluate whether their contribution to the diet could be explained by variation in prey biomass or by changes in selectivity, and (5) analyzed the relationships between relevant biophysical variables and godwit selectivity of earthworms and leatherjackets.

## 2 Methods

### 2.1 Study Area

The study was carried out in southwest Friesland, the Netherlands (52°57′36″ N, 5°27′36″ E), an important breeding area for the continental population of black‐tailed godwits (Kentie et al. 2016). The region consists predominantly of agricultural land used for dairy farming, ranging from traditionally managed (i.e., surface‐drained, herb‐rich meadows with low‐frequency fertilization and mowing regimes) to conventionally managed (i.e., deep‐drained, ryegrass *Lolium perenne* monocultures with high‐frequency fertilization and mowing regimes; Barba‐Escoto et al. 2024; Craft et al. 2022; Howison et al. 2018).

### 2.2 Fecal Sample Collection

Between 10 and 31 March 2022 (pre‐nesting) and 16 May and 30 June 2022 and 2023 (post‐nesting), we collected 249 fecal samples from flock‐foraging godwits on 50 agricultural grassland locations (pre‐nesting 2022: 8 locations; post‐nesting 2022: 19 locations; post‐nesting 2023: 23 locations) throughout the study area. All locations in the pre‐nesting period were unmown grasslands. In the post‐nesting period, of the 42 locations, 39 were recently mown, two had been grazed by cows, and one was not mown. To maximize the likelihood that prey remains in feces were consumed at the location where feces were collected, we gathered feces after observing foraging flocks for at least 30 min. To avoid older feces with deteriorated DNA or feces from other meadow bird species, we collected feces immediately after the godwits left the area. We inspected whether feces were fresh by poking them with a toothpick before collecting them. To avoid cross contamination, fecal samples were collected with one‐use wooden lollypop sticks or metal spoons (cleaned with 70% ethanol in between each sample collection). All samples were stored in 96% ethanol and stored in a freezer at −20°C.

### 2.3 Biophysical Characteristics and Soil Macrofauna Prey Biomass

To measure food biomass at godwit self‐selected foraging sites, we collected six soil macrofauna samples shortly after collecting fecal samples. We used soil cores of 20 × 20 cm width and 10 cm depth, and hand‐collected all specimens into storage tubes. Because in this study, we aimed to collect potential prey availability from the perspective of a godwit, we collected prey items only in the upper 10 cm of the soil (the length of an adult godwit bill; Schroeder et al. 2008), and considered prey items below 10 cm unavailable. In the laboratory, we identified and sorted earthworms to species or genus level. All other macrofauna were identified and sorted either to family (e.g., leatherjackets), order, or class level (following Beintema et al. 1995; Zwarts 1993). We counted all individual specimens and measured the fresh biomass of each specimen, using a scale, to the nearest mg.

To evaluate soil and vegetation characteristics that may affect diet composition and the selectivity for either earthworms or leatherjackets, we measured soil moisture content, soil penetration resistance, and vegetation height (three samples at each soil core). We used a soil moisture meter (model: ML3 ThetaKit Delta‐T Devices) sampling to a depth of 0–5 cm and at a distance of < 10 cm to the edge of the soil core, a soil penetrometer (models: Handpenetrometer voor toplagen—IB, Eijkelkamp and Handpenetrometer Eijkelkamp—1 m), and a ruler fixed on the ground to measure the height of the blade of grass that was closest to the ruler.

### 2.4 DNA Metabarcoding Analyses

We extracted DNA from fecal samples using the Invitrogen PureLink Microbiome DNA Purification kit (Thermo Fisher Scientific, Waltham, Massachusetts, USA), following the protocol in Verkuil et al. (2022). We amplified a 313 bp fragment of the mitochondrial COI gene using the generic metazoan primer pair mlCOIintF and jgHCO2198 (Leray et al. 2013). We used this generic primer because we expected both insects and earthworms as prey taxa. Primers were checked for mismatches against an alignment of COI sequences of expected prey taxa retrieved from GenBank (Benson et al. 2009). We followed the PCR conditions described in Verkuil et al. (2022). PCR products were sequenced on the Illumina MiSeq Sequencer at the Department of Human Genetics, Leiden University Medical Centre, targeting a read depth of 50,000 reads per sample. Sample libraries were prepared using the MiSeq V3 kit (600 cycles), following the standard library preparation protocol of the manufacturer, which involves the second PCR, attaching indices and adapters to the first PCR products.

To correct for potential contamination during field sampling, we collected six negative field controls by either opening and closing the collection tubes in the field or by inserting the collection material (metal or wooden spoon) in the tubes. To examine the risk of contamination by macrofauna inhabiting the surfaces from which we collected feces, we included six surface samples of soil or vegetation collected with a metal or wooden spoon.

For the commonly consumed prey taxa with similarly high frequencies of occurrence (FOO), we chose to compare the relative read abundance (RRA) instead. We also tested how closely RRA potentially reflects the relative biomass of those taxa by composing two mock samples. We tested this only for Insecta and Lumbricidae because they were expected to be important prey items. We consider the outcome a validation for approximating relative biomasses of those prey taxa in the fecal samples. These mock samples also allowed us to confirm that the primers could detect the expected prey taxa. The mock samples consisted of known relative wet weight of insects and earthworms with (1) 80% insect and 20% earthworm, (2) 50% insect and 50% earthworm, and (3) 20% insect and 80% earthworm. In the first mock sample (Batch 1, see Figure 1), *Vespula germanica* was used as insect species and *Lumbricus terrestris* as earthworm species. In the second mock sample (Batch 2, see Figure 1), *Musca domestica* was used as insect and *Aporrectodea caliginosa* as earthworm species. Additionally, eight negative DNA extractions and nine negative PCR controls were included to track possible contamination of reagents.

**FIGURE 1:**
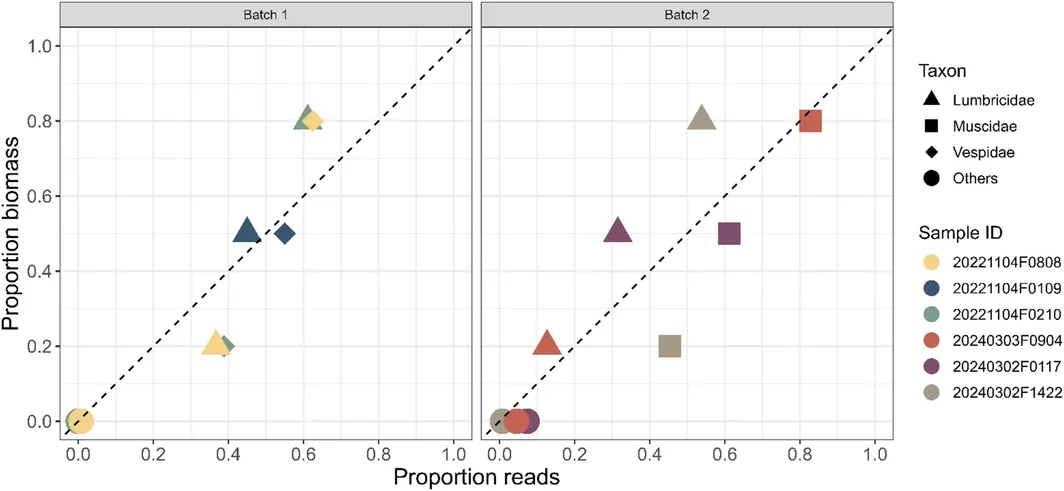
Proportion of biomass of Insecta and Lumbricidae added to the mock samples versus the proportion of DNA sequence reads of Insecta and Lumbricidae in each mock sample. Insecta was represented by *Vespula germanica* and Lumbricidae by *Lumbricus terrestris* (first batch), or by *Musca domestica* and *Aporrectodea caliginosa* , respectively (second batch).

The fecal samples and negative field controls from 2022 (*n* = 108 and *n* = 3, respectively) and 2023 (*n* = 141 and *n* = 3) were sequenced in two separate batches. Both batches included three mock samples, negative extraction controls (*n* = 3 for 2022; *n* = 5 for 2023), and PCR controls (*n* = 2 for 2022; *n* = 7 for 2023). PCR controls from the 2022 batch were pooled before sequencing; PCR controls from the 2023 batch were sequenced separately. To test for within‐batch consistency, we sequenced 10 fecal samples twice in the same batch. To test for between‐batch consistency, we sequenced five fecal samples from the first and second batch in a third batch.

Paired‐end reads were merged and filtered using the software package USEARCH 11 (Edgar 2010). For details on the parameters used for filtering, see Verkuil et al. (2022). Primer sequences were removed using Cutadapt 4.4 (Martin 2011). Filtered reads were clustered into amplicon sequence variants (ASVs) using the UNOISE2 algorithm implemented in USEARCH (Edgar 2016). The ASVs were taxonomically assigned using BLAST+ 2.14 (Camacho et al. 2009), by means of both the core_nt database of GenBank (using a local copy created on 28 January 2025; Benson et al. 2009) and the Barcode of Life Data System (BOLD; using a local copy created on 11 March 2024; Ratnasingham and Hebert 2007) as reference databases. Higher‐level taxonomic assignments were made by assessing the lowest common ancestor of BLAST results (Beentjes et al. 2019). See Appendix S1 for details on the taxonomic assignment.

After the taxonomic assignments of the ASVs, we excluded fecal samples from non‐target host species. Samples were considered to stem from non‐target species when they contained more reads from other bird species than godwits, or when any single non‐godwit bird species had a read count exceeding 80% of the godwit read count (*n* = 17). Then, we selected six classes covering potential prey items for godwits: Insecta, Clitellata, Gastropoda, Arachnida, Diplopoda, and Malacostraca. The final ASV table was corrected for all negative controls (field + extraction + PCR) by subtracting the maximum number of reads for each ASV in the controls from the reads in each sample. Furthermore, we removed samples with less than 30 reads of target taxa because these samples did not adequately pick up the prey diversity in the droppings (Figure S2.1). This resulted in a final number of 207 samples that were analyzed (27 pre‐nesting 2022, 66 post‐nesting 2022, 114 post‐nesting 2023), with 1–9 samples per location.

The taxonomic composition of godwit feces is described based on the RRAs of each family taxon. The RRA of each family is calculated by dividing the number of reads of a family by the total number of reads in a fecal sample. While we recognize that not all taxa found in feces represent primary prey items (see result Section 3.2: “Taxonomic description of fecal samples”), and some may be secondary prey items (parasites or mites), this group of taxa only comprises a small portion of the reads, and therefore, we chose to refer to the taxonomic composition of fecal samples as “diet composition”.

### 2.5 Data Analyses

All data handling, visualization, and statistical analyses, were performed in R (version 4.2.22022‐10‐31, R Core Team 2022). All analyses were conducted at the family level, or to the closest possible taxonomic resolution (i.e., unidentified families of known order or class). To visualize the composition of taxa found in godwit fecal samples in the pre‐nesting period of 2022 and post‐nesting of 2022 and 2023, we computed a bipartite network based on the average RRA of families in each period, using the package “bipartite” (Dormann et al. 2008). To facilitate interpretation, we only portrayed families with an RRA of at least 0.5% in each period. Taxonomic differences between the two periods across both years were visualized through a non‐metric dimensional scaling (NMDS) using a Bray–Curtis dissimilarity matrix with the function *metaMDS* from the “vegan” package. The points in the ordination plot were colored according to period, and 95% confidence ellipses were drawn around group centroids to illustrate the separation between periods and the multivariate dispersion of samples within each period.

When assessing shifts in diet composition between the pre‐ and post‐nesting period, we only considered comparisons within 2022, when both periods were sampled. To quantify the level of dietary niche overlap between the two periods (pre‐ and post‐nesting in 2022), we calculated the Schoener index of overlap using the average RRA of all families in each period (see examples in Coelho et al. 2024). We obtained a mean Schoener overlap index value by bootstrapping 999 times, using the *niche.overlap.boot* function from the package “spaa” (Zhang 2025). Schoener index values range from 0 to 1, with 0–0.39 being considered low, 0.4–0.6 intermediate, and 0.61–1 as high niche overlaps (Grossman 1986; and see examples in Coelho et al. 2024; Maitra et al. 2020). Low overlap values indicate substantial differences in diet composition, and high overlap values represent significant similarities between periods (Grossman 1986).

Differences in diet composition between periods were further tested statistically with a generalized linear model for multivariate data (mGLM), using the *manyglm* function of the package “mvabund” (Wang et al. 2012) and a negative binomial distribution. To account for differences in the number of locations sampled between the two periods, we conducted a power analysis. This consisted in randomly resampling our original data set for five iterations, with reposition, to keep only eight locations out of 19 from the post‐nesting period each time, aiming at comparing them against the same number of locations in the pre‐nesting period. For each iteration, diet composition was compared between periods using a mGLM with a negative binomial distribution. To identify which families explained most of the variation in diet composition between the periods, we ran univariate tests with the function *anova.manyglm*, and *p*‐values were adjusted for multiple testing using a step‐down resampling algorithm (argument *p.uni =* “*adjusted*”; see Wang et al. 2012). We visualized the RRA of the most important taxa in the pre‐ and post‐nesting period with an NMDS. The function *ordisurf* was used to fit smooth surfaces over the ordination plot aiming at assessing and quantifying the gradient of influence of each family across the samples between both periods. Following this, we ran two univariate generalized linear mixed models (uGLMMs) with a binomial family distribution to compare the RRA of the two most important taxa in the godwit fecal samples between the periods. For these models, we used the RRA as response variable (continuous variable ranging from 0 to 1), and included period (categorical with two levels, pre‐ and post‐nesting) as fixed effect. Moreover, we added location ID as a random intercept to account for the lack of independence between samples from the same foraging location. To account for differences in the number of locations sampled between periods, we again randomly resampled the data set five times, with reposition, to select eight out of 19 locations from the post‐nesting period and compared the RRA of the two most important taxa between periods using uGLMMs with a binomial distribution and location ID as a random intercept.

To examine how diet differences were related to biophysical variables of the habitat, namely soil moisture content, soil resistance, and vegetation height, we ran mGLMs with a negative binomial family distribution. Due to the correlation among the biophysical variables (Table S2.1 in Appendix S2), we tested each variable in a separate model. For each variable, we used the *anova.manyglm* function with a likelihood ratio test to compare nested models and assess which one best described the relationship between the biophysical variables and the diet composition in the two periods (Darragh et al. 2020; Zurdo et al. 2023). These models included (1) only period, (2) period and the biophysical variable (soil moisture content (percentage, 0–100), soil resistance (N/cm^2^), or vegetation height (cm)), and (3) the interaction between period and the environmental variable. Subsequently, we used the *summary.manyglm* function to obtain the deviance explained by the environmental variables in the models with the best fit. We visualized the relationships between the environmental variables and taxonomic composition of the fecal samples with a NMDS plot and used the function *ordisurf* to fit smooth surfaces over the ordination plot aiming at interpreting and quantifying the gradient effect of each biophysical variable across the sampling points between periods.

To test the hypothesis that the presence of earthworms and leatherjackets in the feces could be explained by their soil biomass, we first plotted the percentage of DNA reads of both families against their percentage of biomass in the soil at each foraging location. Proportions of earthworms and leatherjackets in the fecal samples and in the soil were calculated from the total number (of DNA reads, or biomass in the soil) of earthworms and leatherjackets combined. Secondly, we examined the selectivity of earthworms and leatherjackets by calculating the Ivlev Electivity Index (Ivlev 1961) for both prey types in each foraging location. The electivity values *E* range from −1.0 to 1.0, with values between −1.0 and −0.3 indicating avoidance or inaccessibility of a taxon, values between −0.3 and 0.3 indicating random selection from the environment, and values between 0.3 and 1.0 indicating positive selection (Qiao et al. 2022; Strauss 1979).

Finally, to examine the relationship between the selectivity of earthworms and leatherjackets and the biophysical habitat characteristics (measured through soil moisture content, soil resistance, and vegetation height), we conducted six Gaussian uGLMMs. Electivity values of earthworms and leatherjackets were used as response variables (continuous values, ranging from −1.0 to 1.0), and for each prey item we ran three models: one with soil moisture content, one with soil resistance, and one with vegetation height as predictors. All models included period and its interaction with the biophysical variable as fixed effects. Post hoc tests were executed to further investigate the effects of the biophysical variables on the selectivity of prey items separately, per period.

## 3 Results

### 3.1 DNA Metabarcoding

There was little overlap in taxa found in negative controls and fecal samples (Tables S2.2–S2.4 of Appendix S2). The most frequently occurring taxa in the fecal samples, Lumbricidae and Tipulidae, accounted for a total of 27 and 31 reads, respectively, across all negative controls. The soil samples (*n* = 6) included 12,697 reads from 56 target taxa, and included 18 Lumbricidae and no Tipulidae reads. For an overview of all taxa occurring in soil samples, see Table S2.5.

The RRA of Insecta and Lumbricidae in the mock samples closely reflected the relative biomass of those taxa included (Figure 1; Figure S2.2, Table S2.6 in Appendix S2). Therefore, we used RRA as an approximation of the taxonomic composition of the fecal samples. Furthermore, the 10 samples re‐sequenced in a third batch showed consistent read abundances across both original batches (Figures S2.3 and S2.4 in Appendix S2), indicating no batch effect. Similarly, duplicate samples within the second batch matched their originals, confirming that sequencing did not affect the relative abundance of taxonomic reads (Figure S2.5 in Appendix S2).

### 3.2 Taxonomic Description of Fecal Samples

In the fecal samples, we found 189 different target taxa belonging to 95 different families (Tables S3.1 and S3.2 in Appendix S3), within the classes Insecta, Clitellata, Arachnida, and Gastropoda (Figure 2). Of the 189 taxa, 77 were identified to the species level. The sequence similarity threshold used for taxonomic assignment was 98%. RRAs were highest for Tipulidae and Lumbricidae (Figure 2; Table S3.1: overview of RRA of all families). Reads of Lumbricidae occurred in all samples in the pre‐nesting period, in 97% of samples post‐nesting 2022, and 39% post‐nesting 2023 (Table S3.2: overview of frequency of occurrence (FOO) of all families). Tipulidae reads occurred in 93% of samples pre‐nesting, 100% of samples post‐nesting 2022, and 97% post‐nesting 2023 (Table S3.2). Reads of spiders Araneae, snails Gastropoda, beetles Coleoptera and butterflies Lepidoptera occurred in smaller amounts in the feces (Figure 2; Table S3.1). However, unidentified Araneae had high FOO in each period (pre‐nesting: 100%, post‐nesting 2022: 95%, post‐nesting 2023: 94%, see Table S3.2).

**FIGURE 2:**
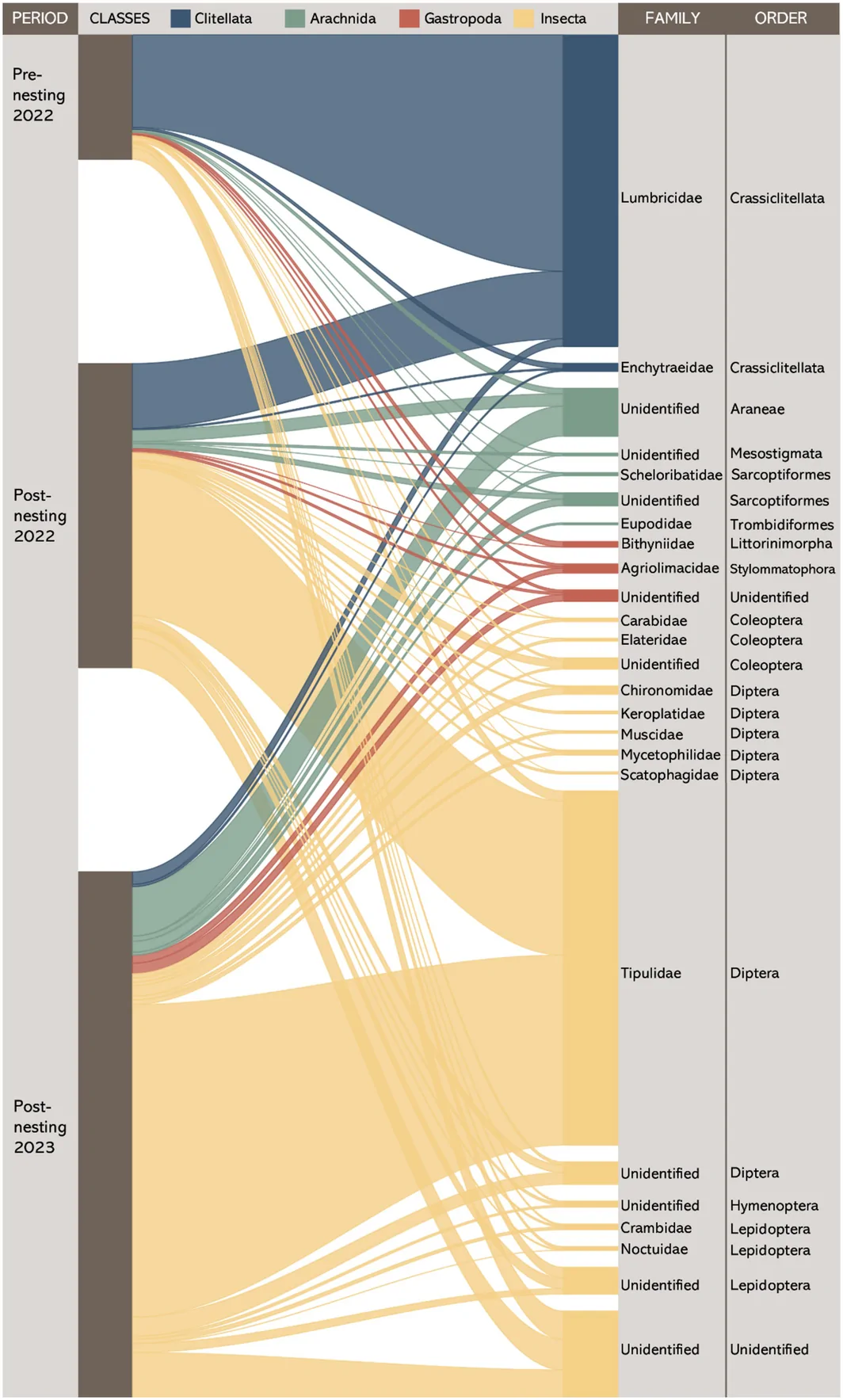
Bipartite network portraying the average relative read abundances of families within the classes Arachnida, Insecta, Clitellata, and Gastropoda found in godwit feces in three periods: Pre‐nesting 2022 (*N* = 27), post‐nesting 2022 (*N* = 66), and post‐nesting 2023 (*N* = 114). The height of period rectangles is scaled to sample size. The 37 prey families shown have a relative read abundance of at least 0.5% in each period. Colors indicate taxonomic classes.

Fecal metabarcoding also detects secondary prey items (species preyed upon by the hosts' prey) and gut parasites, parasitic wasps and mites, such as the Mesostigmata, Sarcoptiformes, and Trombidiformes found in the godwit fecal samples (Figure 2, Tables S3.1 and S3.2). However, the main taxonomic groups found in fecal samples were most likely dietary items (Beintema et al. 1995; Zwarts and Blomert 2010), although it remains uncertain which life stages were consumed. Tipulidae may have been consumed as flying adult crane flies or as leatherjackets. Nonetheless, our foraging observations suggest that the Tipulidae reads found in the feces stem for the greater majority, if not all, from leatherjackets in the soil rather than flying adults. Hence, we used the term “leatherjackets” when referring to Tipulidae.

### 3.3 Differences in Diet Composition Between Periods and Influence of Specific Prey and Habitat Conditions

Changes in diet composition between the pre‐ and post‐nesting period in 2022 were considerable (Figures 2 and 3), as indicated by the intermediate levels of niche overlap (*D* = 0.519, CI = 0.304–0.794; Figure 3), and further supported by a significant difference in diet composition between the two periods (mGLM, deviance = 33.13, *p* = 0.001). Most of these differences were due to the changes in RRA of leatherjackets, followed by that of earthworms (Table S3.3; Figure 2). The contribution of earthworms significantly reduced from 73% ± 26% SD pre‐nesting to 21% ± 26% post‐nesting (Table S3.1, uGLMM: *β* = −2.32, *z*‐ratio = −4.40, *p* < 0.001, Figure 4A), and the contribution of leatherjackets increased significantly from 3% ± 4% to 47% ± 29% (Table S3.1, uGLMM: *β* = 3.35, *z*‐ratio = 2.93, *p* = 0.003; Figure 4A). The results of both the mGLM and uGLMMs were consistent with our findings using the full data set across all iterations of the power analyses (Tables S3.4 and S3.5). Soil moisture content explained most of the seasonal diet variation between the pre‐ and post‐nesting period (Tables 1 and 2). In the pre‐nesting period, the diet composition was associated with moist, soft soils with low vegetation height, whereas post‐nesting diet composition was related to drier, harder soils with higher vegetation (Figure 4B).

**FIGURE 3:**
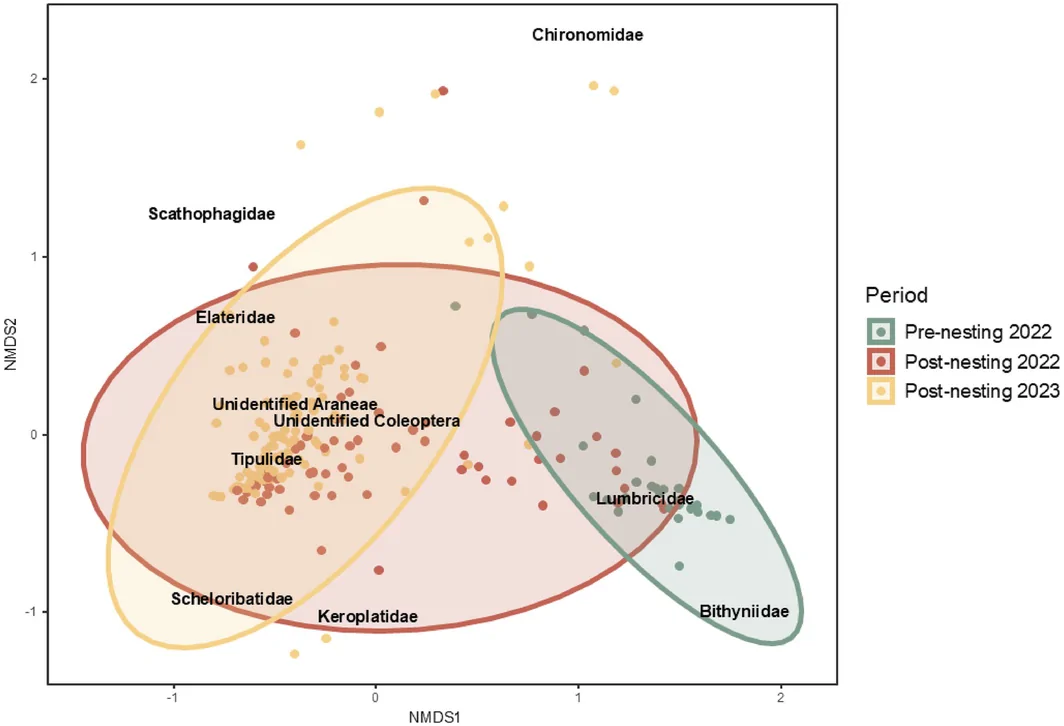
Non‐metric dimensional scaling (NMDS) of taxonomic composition of families within Arachnida, Insecta, Clitellata, and Gastropoda found in godwit feces, calculated using the Bray–Curtis dissimilarity index on relative read abundances of families identified in fecal samples (*k* = 2, stress = 0.13). Ellipses represent a 95% confidence level. The 10 families with highest deviance values are plotted at their centroids. Data of 2 years (2022 and 2023) are visualized here, but formal analyses have been conducted only on data from the pre‐ and post‐nesting periods of 2022.

**FIGURE 4:**
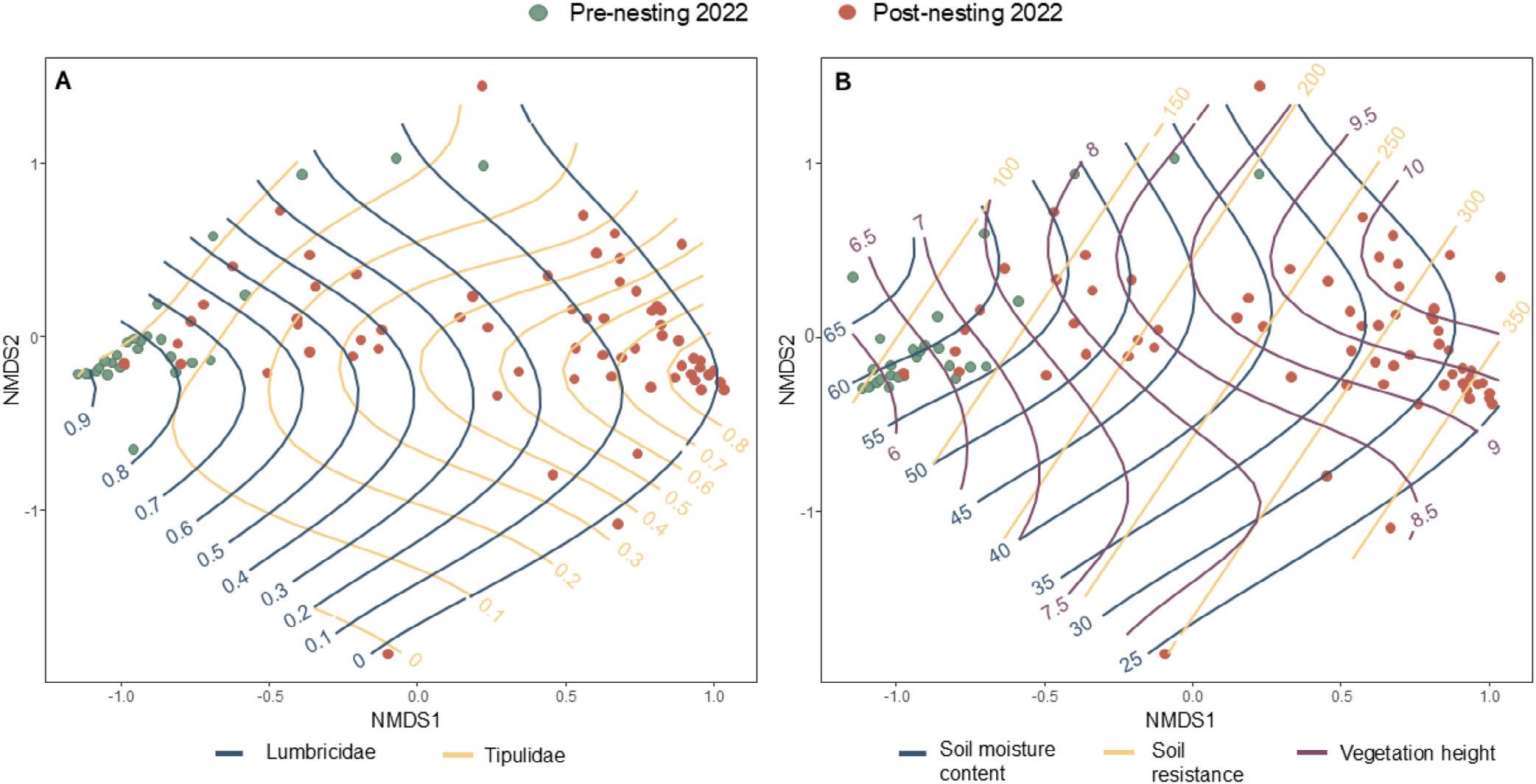
Non‐metric dimensional scaling plots of taxonomic composition pre‐ and post‐nesting, with (A) the relative read abundances of earthworms Lumbricidae and leatherjackets Tipulidae and (B) the environmental variables soil moisture content (%), soil resistance (N/cm^2^) and vegetation height (cm) fitted as smooth surfaces.

**TABLE 1:** Analysis of deviance for mGLMs based on likelihood ratio tests (LRTs) testing the influence of biophysical habitat variables on diet composition in pre‐ and post‐nesting period in 2022.

Models	Δ Deviance	Residual DF	*p*
Soil moisture content			
Period		89	
Period + Soil moisture content	12.47	88	0.046
Period*Soil moisture content	**5.41**	**87**	**0.048**
Soil resistance			
Period		89	
**Period + Soil resistance**	**11.962**	**88**	**0.055**
Period*Soil resistance	2.943	87	0.142
Vegetation height			
**Period**		**89**	
Period + Vegetation height	5.159	88	0.364
Period*Vegetation height	1.915	87	0.160

*Note:* Each model is compared to the model above it. Best models (based on LRT results) are highlighted in bold.

**TABLE 2:** Best models (from model selection in Table 1) describing the influence of biophysical habitat variables on diet composition in the pre‐ and post‐nesting period in 2022.

Models	LR	*p*
Soil moisture content		
Intercept	80.43	0.001
Period	8.47	0.063
Soil moisture content	15.78	0.050
Period*Soil moisture content	5.41	0.119
Resistance		
Intercept	105.69	0.001
Period	14.47	0.020
Resistance	11.96	0.200

### 3.4 Influence of Seasonal Variation in Prey Availability and Habitat Conditions on Prey Selectivity

In the pre‐nesting period, godwits were generally feeding on earthworms as much as expected based on earthworm biomass in the soil (Figures 5 and 6). The average proportion of earthworms in the soil decreased from 0.91 ± 0.14 pre‐nesting to 0.58 ± 0.23 post‐nesting, but the decrease in earthworms RRAs found in the feces was stronger (0.93 ± 0.13 pre‐nesting to 0.34 ± 0.30 post‐nesting; Figure 5). This aligned with the electivity values of earthworms, which changed from between −0.3 and 0.3 in all locations in the pre‐nesting period (indicating random selection from the soil), to below −0.3 in 9 of 19 locations in the post‐nesting period, suggesting avoidance or inaccessibility (Figure 6). In the case of leatherjackets, in the pre‐nesting period they were either selected (1 location), avoided (4 locations) or randomly selected from the soil (2 locations), while during post‐nesting, leatherjackets were mostly found as much as expected based on their biomass in the soil (12 locations), or otherwise selected (7 locations). Post‐nesting, the selectivity for earthworms decreased when the soil moisture content was lower and soil resistance was higher (Figure 7, Table 3; Table S3.6). Conversely, the selectivity for leatherjackets was not related to soil moisture content or soil resistance, but increased with vegetation height in the post‐nesting period (Figures 6 and 7, Table 3; Table S3.6).

**FIGURE 5:**
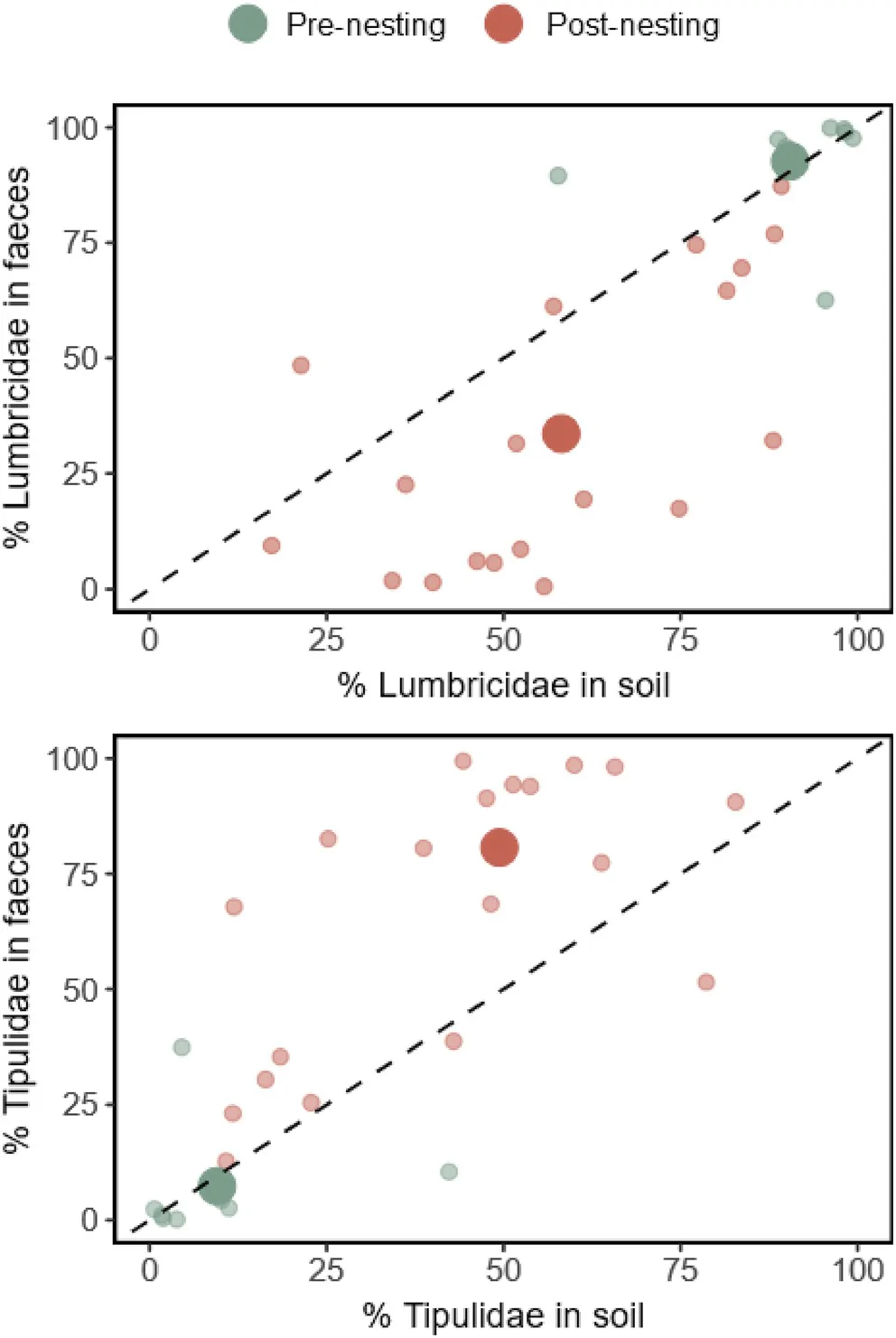
The % read abundance of earthworms Lumbricidae and leatherjackets Tipulidae detected in feces against their % biomass in the soil in the pre‐nesting (green) and post‐nesting (red) period. Percentages were calculated from the total number (DNA reads in feces, or biomass in the soil) of Lumbricidae and Tipulidae combined. Large circles reflect the averages within each period. Points along, above, or below the dashed *x* = *y* line indicate locations where the proportion of Lumbricidae or Tipulidae reads matched, were more abundant, or less abundant than their proportional biomass in the soil, respectively.

**FIGURE 6:**
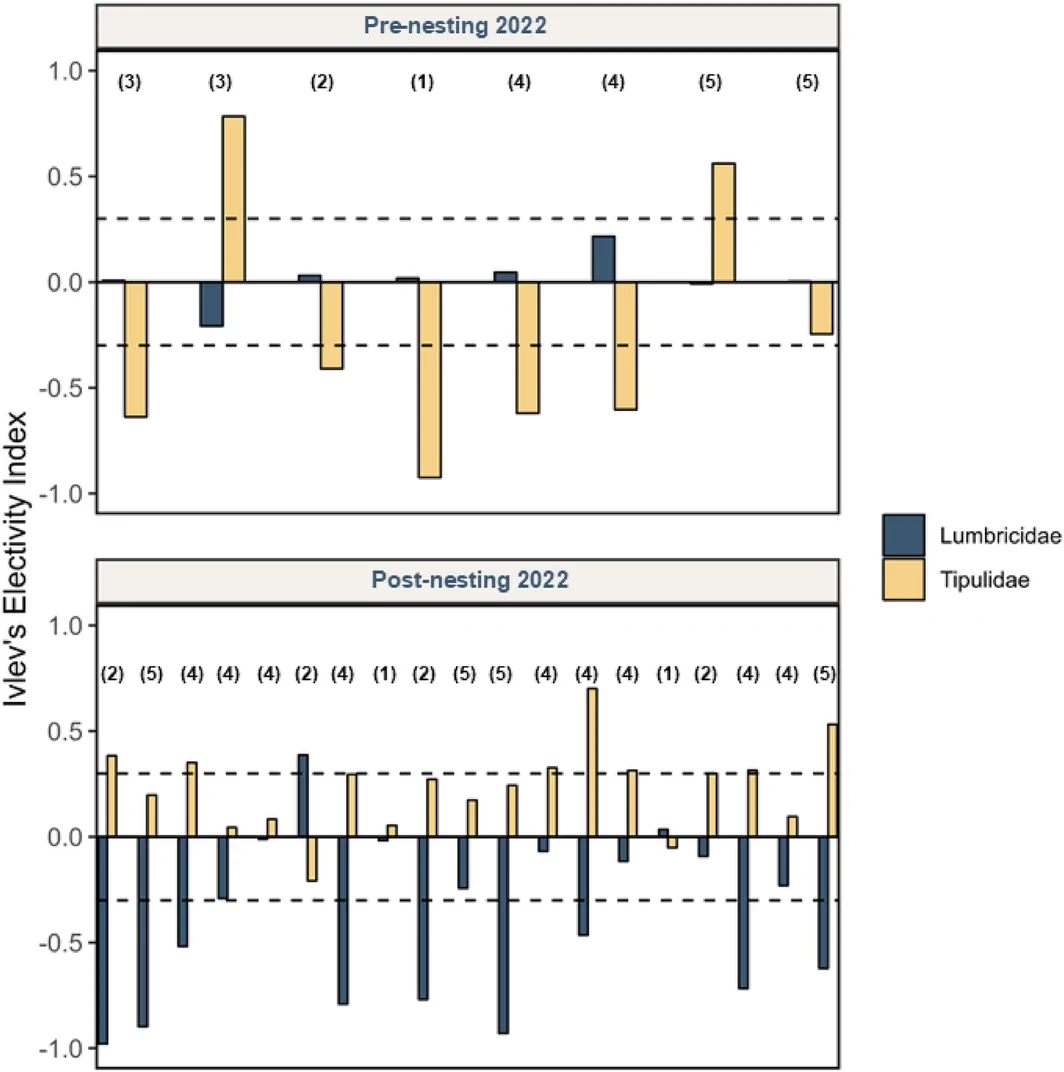
Ivlev Electivity Index for earthworms Lumbricidae (blue) and leatherjackets Tipulidae (yellow) in each godwit foraging location, pre‐ and post‐nesting in 2022. Index values between, below, and above dashed lines indicate random selection, avoidance/inaccessibility, and selection from the environment, respectively. Numbers within brackets indicate the number of fecal samples collected at that location.

**FIGURE 7:**
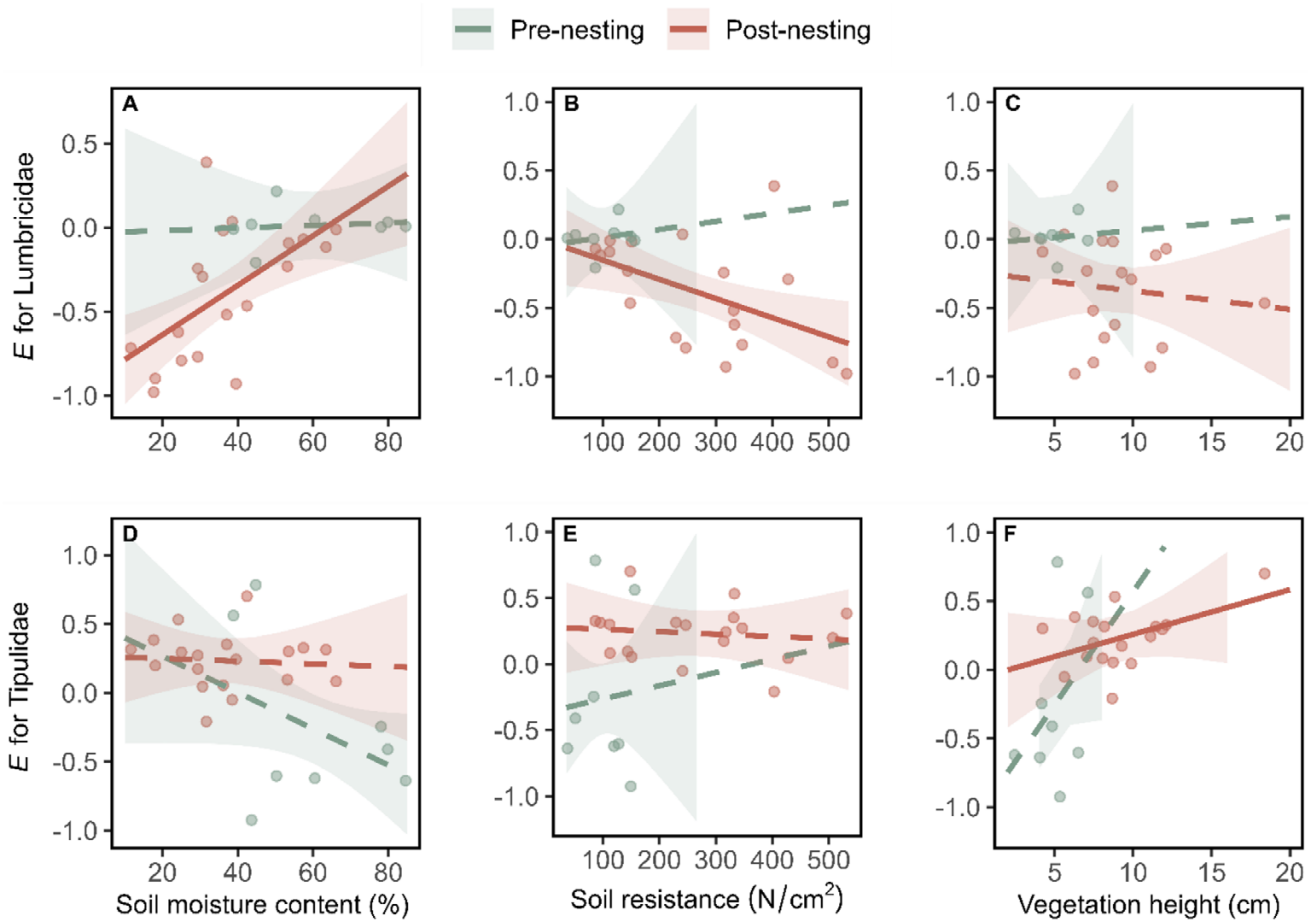
Predicted relationships between the Ivlev's Electivity Index for Lumbricidae and (A) soil moisture content, (B) soil resistance, and (C) vegetation height, and the same relationships for Tipulidae and (D) soil moisture content, (E) soil resistance, and vegetation height (F), based on the GLMMs described in Table 3. Colored lines represent fitted values in the pre‐nesting (green) and post‐nesting (red) period and shaded ribbons indicate 95% confidence intervals.

**TABLE 3:** uGLMMs investigating the relationships between the Ivlev Electivity Index of earthworms and leatherjackets, and biophysical habitat conditions, in the pre‐ and post‐nesting period 2022.

	Estimate	SE	*z*	*p*
**Lumbricidae**				
Soil moisture content				
(Intercept)	−0.933	0.174	−5.360	**< 0.001**
Soil moisture content	0.015	0.004	3.396	**< 0.001**
Period: Pre‐nesting	0.900	0.409	2.200	**0.028**
Soil moisture content*Period: Pre‐nesting	−0.014	0.007	−1.902	0.057
Soil resistance				
(Intercept)	−0.013	0.157	−0.081	0.936
Soil resistance	−0.001	0.001	−2.67	**0.008**
Period: Pre‐nesting	−0.033	0.33	−0.101	0.920
Soil resistance*Period: Pre‐nesting	0.002	0.003	0.734	0.463
Vegetation height				
(Intercept)	−0.242	0.259	−0.935	0.35
Vegetation height	−0.013	0.027	−0.499	0.618
Period: Pre‐nesting	0.206	0.531	0.388	0.698
Vegetation height*Period: Pre‐nesting	0.023	0.094	0.249	0.804
**Tipulidae**				
Soil moisture content				
(Intercept)	0.269	0.217	1.241	0.215
Soil moisture content	−0.001	0.005	−0.181	0.856
Period: Pre‐nesting	0.262	0.51	0.513	0.608
Soil moisture content*Period: Pre‐nesting	−0.012	0.009	−1.335	0.182
Soil resistance				
(Intercept)	0.282	0.194	1.451	0.147
Soil resistance	0	0.001	−0.283	0.777
Period: Pre‐nesting	−0.645	0.409	−1.579	0.114
Soil resistance*Period: Pre‐nesting	0.001	0.003	0.353	0.724
Vegetation height				
(Intercept)	−0.066	0.265	−0.251	0.802
Vegetation height	0.032	0.027	1.184	0.236
Period: Pre‐nesting	−1.01	0.543	−1.861	0.063
Vegetation height*Period: Pre‐nesting	0.132	0.096	1.372	0.170

*Note:* The post‐nesting period is the reference value for the comparisons. Bold values indicate significant relationships, with the threshold for significance set at α = 0.05.

## 4 Discussion

This study provides the first detailed diet description, inferred by DNA metabarcoding of feces, of adult continental black‐tailed godwits. The diet of godwits foraging in flocks on grassland soils mostly comprised earthworms and leatherjackets, but changed considerably from the pre‐nesting to the post‐nesting period. Between arrival on their breeding grounds and the initiation of clutches (March; characterized by moist, soft soils), godwits fed mostly on earthworms, approximately as expected given the soil's earthworm biomass in this period. In contrast, after nesting (end of May and June; characterized by generally drier and harder soils) godwits switched to a diet dominated by leatherjackets, while consuming earthworms noticeably less than expected given their soil biomass. Although in this study, sample sizes differed between periods, our power analysis showed that the dietary taxa shift was consistent and not biased by sample size differences. We are aware of the hesitation to use RRA of the taxa detected to quantify their biomass contribution (see Deagle et al. 2019). However, since we contrasted just two classes, Insecta or Clitellata, for which our mock samples suggested that biases caused by primer choice and PCR were small, and since we accounted for contamination, we used RRA as an approximation of relative consumed biomass. The electivity of earthworms was positively affected by soil moisture, and negatively by soil resistance, but only in the post‐nesting period, while the electivity of leatherjackets was only affected by vegetation height and also exclusively in the post‐nesting period, with a positive albeit weak effect. These results show that habitat conditions, especially those linked with the desiccation and hardening of soils, are important barriers to food accessibility.

In the pre‐nesting period, godwits mainly ate earthworms, as expected (Beintema et al. 1995; Zwarts 1993), which was linked to their high relative biomass in the soil. The smaller contributions of earthworms to the godwit diet in the post‐nesting period coincided with their declining densities in the soil. However, this did not fully explain why godwits fed less on earthworms in that period. At half of the post‐nesting foraging locations, earthworms had negative electivity values (≥ −0.3), indicating that they were present but consumed only in small quantities or not at all. The finding that electivity values for earthworms decreased with lower soil moisture and higher soil resistance values indicates that in desiccated and hardened soils earthworms became inaccessible or undetectable. A similar pattern has been observed for lapwing *Vanellus vanellus* and redshank *Tringa totanus* breeding on a coastal grazing marsh, who feed less on earthworms toward the end of their breeding season (Ausden et al. 2003). Godwits that hunt for earthworms require soft soils which their bills can penetrate (Kleijn et al. 2011; Veenstra et al. 2026). Moreover, godwits benefit from moist soils that promote earthworm activity and facilitate the transmission of vibrations they induce (du Toit et al. 2024). This enhances the detectability of earthworms through the mechanoreceptors on their bill tips, which godwits are likely to use when foraging for earthworms (Bolze 1968; Cunningham 2010). Leatherjackets, in contrast, live in open‐ended, U‐shaped burrows to depths of 2–4 cm (De Jong 1925), and godwits likely use both tactile and visual cues to locate leatherjackets, making hard and dry soil less of a barrier.

The increased importance of leatherjackets in godwits' diets from pre‐nesting to post‐nesting may be explained by an enhanced profitability of this prey item in grassland soils in June compared to March. The leatherjackets consumed were almost exclusively (90% of RRA) the commonly occurring crane fly species in the Netherlands, *Tipula paludosa* . *T. paludosa* lay their eggs in soils within a few days after emerging between July and September (Coulson 1962; Rennie 1917). Eggs hatch after a fortnight but larvae remain small during autumn and winter (< 1 cm). As the soil warms up in spring, they grow swiftly to a length of 3–4 cm at the end of May (Laughlin 1967). Indeed, the average biomass of individual Tipulidae larvae in March amounted to 80 ± 70 (SD) mg, whereas after breeding this had increased to 360 ± 130 mg. Godwits may have avoided leatherjackets in March that were too small to be profitable, whilst feeding frequently on the larger individuals in May and June. This hypothesis is supported by the FOO of Tipulidae in the pre‐nesting period, which was as high as 93% although the average RRA was only 3%, suggesting that small amounts (i.e., small individuals) of leatherjackets were consumed in this period.

The diet composition shift observed in our study raises questions about which prey items are more profitable, and whether the reduced availability of earthworms in the post‐nesting period forces godwits to forage on less profitable prey items. Zwarts and Blomert (1996) showed that leatherjackets had a higher energy and fat content and lower inorganic loading than earthworms. This may explain why oystercatchers *Haematopus ostralegus* feeding on grasslands during spring in Friesland, the Netherlands, fed more on leatherjackets than on earthworms, even when there were more earthworms present in the soil (Zwarts and Blomert 1996); and it may have played a role in the prey selection in our study as well. Godwits that foraged on Frisian meadows in the 1990s fuelled up quickly on a daily diet of 1100–1300 leatherjackets (Zwarts and Blomert 2010). They increased their body weight by about 10 g per day during breeding, a 3%–4% relative increase (Zwarts and Blomert 2010). This leads us to ask whether the densities of leatherjackets in today's grassland soils enable such fast build‐ups of stores.

In addition to earthworms and leatherjackets, prey taxa that were present in smaller amounts were snails Gastropoda and spiders Araneae. Small quantities of these prey items are also found in the diets of adult lapwing breeding on pastures (Baines 1990). Providing calcium required for eggshell formation and skeletal growth, snails are often eaten by egg‐laying females (Graveland and Berends 1997; Graveland and Gijzen 1994), and by precocial godwit and lapwing chicks (Lagendijk et al. 2025). Similarly, spiders contain high amounts of the amino acid taurine, and positively affect tarsus growth in nestlings (García‐Navas et al. 2013; Samplonius et al. 2016). The influence of taurine on adult birds is less pronounced, but may be connected to migration physiology (Gupta et al. 2020). The high FOO of Araneae in godwit feces in each period may suggest that Araneae contain valuable nutrients that godwits require in small amounts. However, because godwits ate more spiders in the drier post‐nesting period, and especially in June 2023, which was markedly drier than June 2022 (a total of 98.9 mm precipitation in June 2022 vs. 9.3 mm in 2023; KNMI 2026), they may also have served as alternative prey in periods when earthworms were unavailable.

This study shows how prey selectivity depends on habitat conditions interacting with the life cycle of prey and, therefore, provides insights in how different climatic conditions may determine the diet composition of adult godwits in their breeding area. It provides a scaffold to now study the feeding ecology of godwits and assess how they sustain their energetic requirements and respond to local changes in habitat and food conditions. The shift in diet composition of flock‐foraging godwits on grassland from the pre‐nesting to the post‐nesting period described in this study suggests that godwits adjust their diet in response to fluctuations in prey harvestability, although it cannot be excluded that altered nutritional requirements in the period before the journeys to non‐breeding residency sites may have impacted prey choices. Nonetheless, with climate change, exacerbated by intensive agricultural land use, leading to increased soil degradation and desiccation (Kraamwinkel et al. 2021), the question is whether godwits will, in the future, still be able to fuel up on their natal grasslands, or need to deviate to other habitats.

## Author Contributions

**Renée Veenstra:** conceptualization (equal), data curation (lead), formal analysis (lead), investigation (lead), methodology (lead), visualization (lead), writing – original draft (lead). **Yvonne Verkuil:** conceptualization (equal), formal analysis (equal), methodology (equal), validation (equal), writing – review and editing (equal). **Gerrit Potkamp:** formal analysis (equal), methodology (equal), validation (equal), writing – review and editing (equal). **Jeroen Onrust:** conceptualization (equal), supervision (equal), writing – review and editing (equal). **Matty P. Berg:** conceptualization (equal), supervision (equal), writing – review and editing (equal). **Theunis Piersma:** conceptualization (equal), funding acquisition (equal), project administration (equal), supervision (equal), writing – review and editing (equal). **Mohamed Henriques:** conceptualization (equal), formal analysis (equal), methodology (equal), supervision (equal), validation (equal), writing – review and editing (equal).

## Funding

This work was supported by the Dutch Ministry of Agriculture, Fisheries, Food Security and Nature; Vogelbescherming Nederland; and the Provinces of Fryslân, Overijssel, Groningen, Gelderland, Utrecht, Noord‐Holland, and Zuid‐Holland. It was also funded by the LIFE IP program through LIFE IP GrassBirdHabitats (LIFE19 IPE/DE/000004).

## Disclosure

Statement on Inclusion: Our study was conducted by scientists based in the country where the study was carried out and also includes scientists from other countries to ensure a diverse set of perspectives.

## Conflicts of Interest

The authors declare no conflicts of interest.

## Supporting information


**Appendix S1:** ece374495‐sup‐0001‐AppendixS1.docx.

**Appendix S2:** ece374495‐sup‐0002‐AppendixS2.docx.

**Appendix S3:** ece374495‐sup‐0003‐AppendixS3.docx.

## Data Availability

The data for this study will, upon publication, be made publicly available on Dryad: https://doi.org/10.5061/dryad.2rbnzs867.
