# Supplementary material for: Soil Moisture and Prey Life Cycles Promote Seasonal Dietary Shift in a Grassland‐Breeding Migratory Shorebird
Source: Ecol Evol. 2026 Oct 11;16(10):e74495. doi: 10.1002/ece3.74495 (PMC13655214; doi:10.1002/ece3.74495)
Supplement: Supplementary file 1 [file ECE3-16-e74495-s001.docx]

Appendix S2

**From:** Soil moisture and prey life cycles promote seasonal dietary shift in a grassland-breeding migratory shorebird species

*Summary sequencing results*

After correction of reads occurring in field, extraction, and PCR controls, faecal samples contained a total of 3,190,013 reads, with 1,641,482 reads assigned to Animalia. Filtering for potential prey taxa (target taxa from here on) and excluding samples with fewer than 30 reads of target taxa resulted in 161,887 reads. In the PCR controls (n=10) we found 454 reads of target taxa with a maximum of 244 per ASV (Table S2.2). In the extraction (n=8) and field controls (n=7), we found 3670 and 486 reads of target taxa with a maximum of 3015 and 227 reads per ASV, respectively (Tables S2.3 and S2.4).

**Table S2.1.** Spearman rank correlations between environmental variables.

|  | Rho | P value |
| --- | --- | --- |
| Soil moisture ~ Soil resistance | -0.892 | **<0.001** |
| Soil moisture ~ Vegetation height | -0.426 | **0.031** |
| Soil resistance ~ Vegetation height | 0.395 | **0.047** |

**Figure S2.1** Correlation between the number of DNA sequence reads of taxa classes Arachnida, Clitellata, Diplopoda, Gastropoda, Insecta and Malacostraca, in fecal samples and Shannon diversity of these taxa. The vertical line is set at N = 30.

**Table S2.2** Overview of taxa within classes Arachnida, Clitellata, Diplopoda, Gastropoda, Insecta and Malacostraca, detected in PCR controls, continues on next page

|  |  |  | Barcode IDs 2022 | | Barcode IDs 2023 | | |
| --- | --- | --- | --- | --- | --- | --- | --- |
| **Class** | **Order** | **Taxon** | 20221101F1617 | 20221104F1515 | 20240303F1009 | 20240301F0809 | 20240301F1114 |
| Arachnida | Araneae | Nesticidae | 0 | 0 | 0 | 0 | 0 |
|  | Sarcoptiformes | *Dermatophagoides pteronyssinus* | 0 | 0 | 244 | 0 | 0 |
| Clitellata | Crassiclitellata | Aporrectodea | 1 | 0 | 0 | 0 | 0 |
|  |  | *Allolobophora chlorotica* | 0 | 0 | 1 | 0 | 0 |
|  |  | *Aporrectodea caliginosa* | 0 | 0 | 0 | 0 | 0 |
|  |  | *Lumbricus rubellus* | 3 | 0 | 0 | 0 | 0 |
| Insecta | Coleoptera | Coleoptera | 0 | 0 | 0 | 0 | 1 |
|  |  | *Sitona sulcifrons* | 0 | 0 | 1 | 0 | 0 |
|  |  | *Rhinoncus pericarpius* | 0 | 0 | 1 | 0 | 0 |
|  | Diptera | *Botanophila striolata* | 48 | 0 | 0 | 0 | 0 |
|  |  | Culicidae | 0 | 0 | 0 | 0 | 0 |
|  |  | *Desmometopa varipalpis* | 0 | 0 | 0 | 0 | 0 |
|  |  | *Dohrniphora cornuta* | 0 | 0 | 0 | 0 | 29 |
|  |  | *Musca domestica* | 0 | 0 | 0 | 0 | 0 |
|  |  | *Scatella paludum* | 0 | 0 | 1 | 0 | 0 |
|  |  | *Symplecta stictica* | 0 | 0 | 5 | 0 | 0 |
|  |  | Tipula | 0 | 0 | 0 | 0 | 1 |
|  |  | *Tipula paludosa* | 1 | 0 | 1 | 1 | 1 |
|  | Hemiptera | *Anoscopus serratulae* | 0 | 0 | 1 | 0 | 0 |
|  | Unidentified | Insecta | 0 | 0 | 0 | 0 | 0 |
|  |  | **Total of all reads** | 53 | 0 | 255 | 1 | 32 |

**Table S2.2, continued.** Overview of taxa within classes Arachnida, Clitellata, Diplopoda, Gastropoda, Insecta and Malacostraca, detected in PCR controls.

|  |  |  | Barcode IDs 2023 | | | | |  |
| --- | --- | --- | --- | --- | --- | --- | --- | --- |
| **Class** | **Order** | **Taxon** | 20240301F1406 | 20240302F0306 | 20240302F0622 | 20240302F0809 | 20241205F0524 | **Total** |
| Arachnida | Araneae | Nesticidae | 0 | 15 | 10 | 0 | 0 | 25 |
|  | Sarcoptiformes | *Dermatophagoides pteronyssinus* | 0 | 0 | 0 | 0 | 0 | 244 |
| Clitellata | Crassiclitellata | *Aporrectodea* | 0 | 0 | 0 | 0 | 0 | 1 |
|  |  | *Allolobophora chlorotica* | 0 | 0 | 0 | 0 | 0 | 1 |
|  |  | *Aporrectodea caliginosa* | 0 | 0 | 8 | 0 | 0 | 8 |
|  |  | *Lumbricus rubellus* | 0 | 0 | 0 | 0 | 0 | 3 |
| Insecta | Coleoptera | Coleoptera | 0 | 0 | 0 | 0 | 0 | 1 |
|  |  | *Sitona sulcifrons* | 0 | 0 | 0 | 0 | 0 | 1 |
|  |  | *Rhinoncus pericarpius* | 0 | 0 | 0 | 0 | 0 | 1 |
|  | Diptera | *Botanophila striolata* | 0 | 0 | 0 | 0 | 0 | 48 |
|  |  | Culicidae | 0 | 54 | 0 | 0 | 0 | 54 |
|  |  | *Desmometopa varipalpis* | 0 | 0 | 22 | 0 | 0 | 22 |
|  |  | *Dohrniphora cornuta* | 0 | 0 | 0 | 0 | 0 | 29 |
|  |  | *Musca domestica* | 0 | 0 | 2 | 0 | 0 | 2 |
|  |  | *Scatella paludum* | 0 | 0 | 0 | 0 | 0 | 1 |
|  |  | *Symplecta stictica* | 0 | 0 | 0 | 0 | 0 | 5 |
|  |  | *Tipula* | 0 | 0 | 0 | 0 | 0 | 1 |
|  |  | *Tipula paludosa* | 0 | 0 | 0 | 0 | 0 | 4 |
|  | Hemiptera | *Anoscopus serratulae* | 0 | 0 | 0 | 0 | 0 | 1 |
|  | Unidentified | Insecta | 1 | 0 | 0 | 1 | 0 | 2 |
|  |  | **Total of all reads** | 1 | 69 | 42 | 1 | 0 | 454 |

**Table S2.3** Overview of taxa within the classes Arachnida, Clitellata, Diplopoda, Gastropoda, Insecta and Malacostraca, that were detected in extraction controls, continuous on the next page.

|  |  |  | Barcode IDs 2022 | | |
| --- | --- | --- | --- | --- | --- |
| **Class** | **Order** | **Taxon** | 20221101F1221 | 20221104F0101 | 20221104F1414 |
| Arachnida | Araneae | Araneae | 0 | 0 | 0 |
|  |  | Nesticidae | 0 | 0 | 0 |
|  | *Mesostigmata* | *Dermanyssus longipes* | 0 | 0 | 3015 |
|  | *Trombidiformes* | *Demodex folliculorum* | 282 | 0 | 0 |
| *Clitellata* | *Crassiclitellata* | *Allolobophora chlorotica* | 0 | 0 | 0 |
|  |  | *Aporrectodea caliginosa* | 0 | 0 | 0 |
| Gastropoda | *Unidentified* | Gastropoda | 0 | 0 | 0 |
| Insecta | *Coleptera* | *Tanymecus palliatus* | 0 | 0 | 0 |
|  | *Diptera* | *Musca domestica* | 0 | 0 | 0 |
|  |  | Muscidae | 0 | 0 | 0 |
|  |  | Culicidae | 0 | 0 | 0 |
|  |  | Diptera | 0 | 0 | 0 |
|  |  | *Tipula paludosa* | 0 | 0 | 0 |
|  | Hymenoptera | Apidae | 0 | 0 | 0 |
|  | Unidentified | Insecta | 0 | 0 | 0 |
|  |  | **Total of all reads** | 282 | 0 | 3015 |

**Table S2.3, continued.** Overview of taxa within the classes Arachnida, Clitellata, Diplopoda, Gastropoda, Insecta and Malacostraca that were detected in extraction controls.

|  |  |  | Barcode IDs 2023 | | | | |  |
| --- | --- | --- | --- | --- | --- | --- | --- | --- |
| **Class** | **Order** | **Taxon** | 20240301F0917 | 20240302F1010 | 20240303F0910 | 20240301F0504 | 20240302F0520 | **Total** |
| Arachnida | Araneae | Araneae | 0 | 0 | 1 | 0 | 0 | 1 |
|  |  | Nesticidae | 0 | 0 | 154 | 0 | 0 | 154 |
|  | *Mesostigmata* | *Dermanyssus longipes* | 0 | 0 | 0 | 0 | 0 | 3015 |
|  | *Trombidiformes* | *Demodex folliculorum* | 0 | 0 | 0 | 0 | 0 | 282 |
| *Clitellata* | *Crassiclitellata* | *Allolobophora chlorotica* | 0 | 0 | 1 | 0 | 0 | 1 |
|  |  | *Aporrectodea caliginosa* | 0 | 0 | 3 | 0 | 0 | 3 |
| Gastropoda | *Unidentified* | Gastropoda | 0 | 0 | 1 | 0 | 0 | 1 |
| Insecta | *Coleoptera* | *Tanymecus palliatus* | 0 | 0 | 1 | 0 | 0 | 1 |
|  | *Diptera* | *Musca domestica* | 0 | 0 | 52 | 0 | 0 | 52 |
|  |  | Muscidae | 0 | 0 | 1 | 0 | 0 | 1 |
|  |  | Culicidae | 0 | 0 | 133 | 0 | 0 | 133 |
|  |  | Diptera | 0 | 0 | 4 | 0 | 0 | 4 |
|  |  | *Tipula paludosa* | 4 | 0 | 0 | 4 | 4 | 12 |
|  | Hymenoptera | Apidae | 0 | 0 | 0 | 0 | 9 | 9 |
|  | Unidentified | Insecta | 1 | 0 | 0 | 0 | 0 | 1 |
|  |  | **Total of all reads** | 5 | 0 | 351 | 4 | 13 | 3670 |

**Table S2.4.** Overview of taxa within the classes Arachnida, Clitellata, Diplopoda, Gastropoda, Insecta and Malacostraca that were detected in the negative field controls.

|  |  |  | Barcode IDs 2022 | | | Barcode IDs 2023 | | | |  |
| --- | --- | --- | --- | --- | --- | --- | --- | --- | --- | --- |
| **Class** | **Order** | **Taxon** | 20221101F0319 | 20221101F0916 | 20221101F1220 | 20240301F0223 | 20240301F1321 | 20240302F0405 | 20240302F1111 | **Total** |
| Arachnida | Trombidiformes | Bdellidae | 0 | 0 | 0 | 2 | 0 | 0 | 0 | 2 |
| Clitellata | Crassiclitellata | *Allolobophora chlorotica* | 0 | 1 | 0 | 0 | 0 | 0 | 0 | 1 |
|  |  | *Aporrectodea caliginosa* | 0 | 0 | 1 | 0 | 0 | 0 | 0 | 1 |
|  |  | *Lumbricus rubellus* | 0 | 0 | 0 | 0 | 4 | 0 | 1 | 9 |
|  | Enchytraeida | Enchytraeidae | 1 | 0 | 0 | 0 | 0 | 0 | 0 | 2 |
|  | Unidentified | Clitellata | 0 | 1 | 0 | 0 | 0 | 0 | 0 | 1 |
| Gastropoda | Stylommatophora | *Deroceras reticulatum* | 0 | 0 | 0 | 0 | 1 | 0 | 0 | 1 |
| Insecta | Diptera | Culicidae | 0 | 0 | 0 | 0 | 0 | 18 | 0 | 18 |
|  |  | *Dohrniphora cornuta* | 0 | 2 | 0 | 83 | 0 | 0 | 0 | 83 |
|  |  | *Botanophila striolata* | 0 | 0 | 0 | 0 | 227 | 0 | 0 | 227 |
|  |  | *Nephrotoma appendiculata* | 0 | 0 | 0 | 0 | 1 | 2 | 0 | 5 |
|  |  | *Tipula* | 2 | 3 | 0 | 0 | 6 | 0 | 0 | 3 |
|  |  | *Tipula paludosa* | 0 | 0 | 0 | 0 | 0 | 0 | 0 | 11 |
|  | Hymenoptera | Apidae | 0 | 0 | 0 | 0 | 0 | 117 | 0 | 117 |
|  |  | Hymenoptera | 2 | 0 | 0 | 0 | 0 | 1 | 0 | 1 |
|  | Lepidoptera | Lepidoptera | 3 | 6 | 0 | 0 | 0 | 0 | 0 | 1 |
|  | Unidentified | Insecta | 0 | 0 | 1 | 0 | 0 | 0 | 0 | 3 |
|  |  | **Total of all reads** | 8 | 13 | 2 | 85 | 239 | 138 | 1 | 486 |

**Table S2.5** Overview of within the classes Arachnida, Clitellata, Diplopoda, Gastropoda, Insecta and Malacostraca detected in soil samples.

|  |  |  | 2022 | | 2023 | | | |  |
| --- | --- | --- | --- | --- | --- | --- | --- | --- | --- |
| **Class** | **Order** | **Taxa** | 20221101F0223 | 20221101F0412 | 20240302F0614 | 20240303F0908 | 20240301F0108 | 20240302F1312 | **Total** |
| Arachnida | Araneae | Araneae | 1 | 42 | 3 | 7 | 14 | 2 | 69 |
|  |  | *Dicymbium nigrum* | 0 | 0 | 0 | 0 | 0 | 80 | 80 |
|  | Ixodida | Ixodida | 0 | 0 | 0 | 0 | 9 | 0 | 9 |
|  | Mesostigmata | Mesostigmata | 0 | 0 | 0 | 6 | 0 | 8 | 14 |
|  |  | Parasitidae | 0 | 12 | 0 | 0 | 0 | 0 | 12 |
|  | Sarcoptiformes | Chamobatidae | 0 | 0 | 0 | 0 | 0 | 26 | 26 |
|  |  | Sarcoptiformes | 0 | 0 | 0 | 0 | 0 | 39 | 39 |
|  |  | Scheloribates | 0 | 0 | 0 | 0 | 0 | 272 | 272 |
|  |  | Scheloribatidae | 0 | 0 | 0 | 0 | 0 | 47 | 47 |
|  |  | *Liebstadia similis* | 0 | 15 | 0 | 0 | 0 | 0 | 15 |
|  |  | Galumna | 0 | 0 | 0 | 0 | 0 | 3172 | 3172 |
|  |  | Galumnidae | 0 | 0 | 0 | 0 | 0 | 22 | 22 |
|  | Scorpiones | Scorpiones | 1 | 0 | 3 | 0 | 1 | 0 | 5 |
|  | Trombidiformes | Tarsonemidae | 0 | 0 | 0 | 0 | 0 | 1 | 1 |
|  |  | Trombidiformes | 0 | 5 | 4 | 0 | 0 | 3 | 12 |
|  | Unidentified | Arachnida | 14 | 1 | 0 | 0 | 5 | 0 | 20 |
| Clitellata | Enchytraeida | Enchytraeidae | 220 | 0 | 0 | 0 | 1 | 0 | 221 |
|  |  | Enchytraeus | 858 | 0 | 0 | 0 | 0 | 0 | 858 |
|  |  | Fridericia | 0 | 6 | 0 | 1 | 0 | 0 | 7 |
|  |  | Henlea | 1809 | 0 | 0 | 0 | 0 | 0 | 1809 |
|  |  | *Henlea perpusilla* | 0 | 0 | 13 | 0 | 0 | 0 | 13 |
|  | Lumbricidae | *Eiseniella tetraedra* | 0 | 0 | 1 | 0 | 0 | 0 | 1 |
|  |  | *Lumbricus castaneus* | 2 | 0 | 0 | 0 | 0 | 0 | 2 |
|  |  | *Lumbricus rubellus* | 0 | 1 | 0 | 1 | 0 | 0 | 2 |
|  |  | *Satchellius mammalis* | 1 | 0 | 0 | 0 | 12 | 0 | 13 |
|  | Unidentified | Clitellata | 0 | 0 | 179 | 0 | 0 | 0 | 179 |
| Gastropoda | Acochlidiacea | Acochlidiacea | 0 | 0 | 0 | 1 | 0 | 0 | 1 |
|  | Nudibranchia | Nudibranchia | 16 | 1 | 0 | 0 | 1 | 12 | 30 |
|  | Stylommatophora | *Deroceras reticulatum* | 0 | 1 | 0 | 0 | 1 | 0 | 2 |
|  | Systellommatophora | Systellommatophora | 1 | 0 | 0 | 0 | 0 | 3 | 4 |
|  | Unidentified | Gastropoda | 0 | 6 | 0 | 13 | 4 | 0 | 23 |
| Insecta | Coleoptera | Amischa analis | 0 | 0 | 0 | 0 | 3 | 0 | 3 |
|  |  | Coleoptera | 0 | 7 | 6 | 2 | 3 | 7 | 25 |
|  |  | *Philonthus carbonarius* | 0 | 628 | 0 | 0 | 0 | 0 | 628 |
|  |  | *Tanymecus palliatus* | 0 | 0 | 0 | 1 | 0 | 0 | 1 |
|  |  | *Tytthaspis sedecimpunctata* | 0 | 0 | 0 | 0 | 0 | 5 | 5 |
|  | Diptera | Cecidomyiidae | 0 | 0 | 0 | 0 | 270 | 4 | 274 |
|  |  | Chironomidae | 0 | 0 | 0 | 0 | 1 | 0 | 1 |
|  |  | Diptera | 2 | 1 | 1 | 2 | 6 | 0 | 12 |
|  |  | Musca | 0 | 0 | 0 | 1 | 0 | 0 | 1 |
|  |  | *Musca domestica* | 0 | 0 | 0 | 3 | 0 | 0 | 3 |
|  |  | Mycetophilidae | 0 | 0 | 0 | 0 | 29 | 0 | 29 |
|  |  | *Paraphaenocladius impensus* | 10 | 0 | 0 | 0 | 0 | 0 | 10 |
|  |  | Syrphidae | 0 | 0 | 0 | 1 | 1 | 0 | 2 |
|  | Hemiptera | Anoscopus | 0 | 0 | 0 | 0 | 0 | 705 | 705 |
|  |  | *Anoscopus flavostriatus* | 0 | 0 | 0 | 0 | 0 | 83 | 83 |
|  |  | *Anoscopus serratulae* | 0 | 0 | 0 | 0 | 0 | 3533 | 3533 |
|  |  | Cicadellidae | 0 | 0 | 0 | 0 | 0 | 3 | 3 |
|  |  | Hemiptera | 0 | 0 | 0 | 1 | 7 | 0 | 8 |
|  | Hymenoptera | Braconidae | 0 | 5 | 0 | 0 | 5 | 0 | 10 |
|  |  | Platygastridae | 0 | 0 | 0 | 0 | 0 | 7 | 7 |
|  |  | Hymenoptera | 79 | 3 | 0 | 0 | 2 | 3 | 87 |
|  | Lepidoptera | Geometridae | 0 | 0 | 0 | 0 | 0 | 16 | 16 |
|  |  | Lepidoptera | 0 | 28 | 3 | 20 | 30 | 0 | 81 |
|  | Trichoptera | Trichoptera | 0 | 0 | 0 | 1 | 1 | 0 | 2 |
|  | Unidentified | Insecta | 26 | 34 | 9 | 39 | 69 | 11 | 188 |
|  |  | **Total of all reads** | 3040 | 796 | 222 | 100 | 475 | 8064 | 12697 |

**Table S2.6** Number of DNA sequence reads per taxa in each mock sample. Only taxa within the classes Arachnida, Clitellata, Diplopoda, Gastropoda, Insecta and Malacostraca were selected and negative controls PCR and extraction controls were subtracted.

|  | Barcode IDs Batch 1 | | | Barcode IDs Batch 2 | | |
| --- | --- | --- | --- | --- | --- | --- |
|  | 20221104F0808 | 20221104F0109 | 20221104F0210 | 20240303F0904 | 20240302F0117 | 20240302F1422 |
| **Taxon** | Insect : Worm  80 : 20 | Insect : Worm  50 : 50 | Insect : Worm  20 : 80 | Insect : Worm  80 : 20 | Insect : Worm  50 : 50 | Insect : Worm  20 : 80 |
| *Allolobophora* | 0 | 0 | 0 | 0 | 0 | 0 |
| *Allolobophora chlorotica* | 0 | 0 | 0 | 0 | 0 | 0 |
| *Aporrectodea* | 0 | 0 | 0 | 0 | 0 | 0 |
| *Aporrectodea caliginosa* | 0 | 0 | 0 | 2631 | 8148 | 11395 |
| *Aporrectodea longa* | 0 | 0 | 0 | 0 | 0 | 0 |
| *Aporrectodea rosea* | 0 | 0 | 1 | 0 | 0 | 0 |
| *Aporrectodea tuberculata* | 0 | 2 | 2 | 0 | 0 | 0 |
| Diptera | 0 | 0 | 0 | 1 | 7 | 0 |
| *Eurhopalus* | 7 | 0 | 0 | 0 | 0 | 0 |
| *Eurhopalus angustus* | 361 | 19 | 1 | 0 | 0 | 0 |
| Gastropoda | 0 | 0 | 0 | 0 | 0 | 1 |
| Insecta | 0 | 0 | 0 | 941 | 1929 | 169 |
| Lepidoptera | 0 | 0 | 0 | 0 | 0 | 0 |
| Lumbricidae | 0 | 0 | 0 | 0 | 200 | 63 |
| *Lumbricus* | 0 | 0 | 0 | 0 | 0 | 0 |
| *Lumbricus castaneus* | 0 | 0 | 0 | 0 | 0 | 0 |
| *Lumbricus rubellus* | 0 | 0 | 0 | 0 | 0 | 0 |
| *Lumbricus terrestris* | 17865 | 19396 | 18062 | 0 | 0 | 0 |
| *Musca* | 0 | 0 | 0 | 34 | 264 | 1 |
| *Musca domestica* | 0 | 0 | 0 | 17140 | 15866 | 9646 |
| Muscidae | 0 | 0 | 0 | 11 | 81 | 0 |
| *Octolasion* | 0 | 0 | 0 | 0 | 0 | 0 |
| *Octolasion tyrtaeum* | 0 | 0 | 0 | 0 | 0 | 0 |
| *Sargus bipunctatus* | 2 | 0 | 0 | 0 | 0 | 0 |
| *Satchellius* | 0 | 0 | 0 | 0 | 0 | 0 |
| *Satchellius mammalis* | 0 | 0 | 0 | 0 | 0 | 0 |
| *Scaeva pyrastri* | 0 | 0 | 3 | 0 | 0 | 0 |
| Scheloribates | 0 | 0 | 0 | 1 | 0 | 0 |
| *Vespula germanica* | 30418 | 23710 | 11487 | 0 | 0 | 0 |
| **Total of all reads** | 48653 | 43127 | 29556 | 20759 | 26495 | 21275 |

**Figure S2.2** Mock samples from Batch 1 and 2, both ordered from left to right consisting of (1) 80% Insecta and 20% Lumbricidae, (2) 50% Insecta and 50% Lumbricidae, (3) 20% Insecta and 80% earthworm. In the first batch, *Vespula germanica* was used as an insect and *Lumbricus terrestris* as an earthworm Lumbricidae, in the second on *Musca domestica* as insect and *Aporrectodea caliginosa* as an earthworm. The number of target reads with PCR and extraction controls subtracted are indicated for each mock sample on the plot.

**Figure S2.3** Relative read abundances of six classes (Arachnida, Clitellata, Diplopoda, Gastropoda, Insecta and Malacostraca) in ten samples and their repeats, which were sequenced in another, separate batch. The field sample ID is specified for each sample and corresponding repeat, as well as the number of reads of all taxa with controls subtracted.

**Figure S2.4** RRA of classes in repeats vs those in the original samples.

**Figure S2.5** Relative read abundances of six classes (Arachnida, Clitellata, Diplopoda, Gastropoda, Insecta and Malacostraca) in ten samples and their duplicates, samples twice within the same batch to test for within-batch consistency. The field sample ID is specified for each sample and corresponding duplicate, as well as the number of reads of all taxa with controls subtracted.
