# Supplementary material for: Soil Moisture and Prey Life Cycles Promote Seasonal Dietary Shift in a Grassland‐Breeding Migratory Shorebird
Source: Ecol Evol. 2026 Oct 11;16(10):e74495. doi: 10.1002/ece3.74495 (PMC13655214; doi:10.1002/ece3.74495)
Supplement: Supplementary file 2 [file ECE3-16-e74495-s002.docx]

Appendix S3

**From:** Soil moisture and prey life cycles promote seasonal dietary shift in a grassland-breeding migratory shorebird species

**Table S3.1.** The average relative read abundance of all families within the classes Arachnida, Clitellata, Diplopoda, Gastropoda, Insecta and Malacostraca, in the period pre-nesting 2022 (n=27 samples), post-nesting 2022 (n=66) and post-nesting 2023 (n=114).

| **Class** | **Order** | **Family** | Pre-nesting **2022** | Post-nesting 2022 **2022** | Post-nesting **2023** |
| --- | --- | --- | --- | --- | --- |
| Arachnida | Araneae | Linyphiidae | 0.000144385 | 0.000000000 | 0.000599476 |
| Arachnida | Araneae | Lycosidae | 0.000087100 | 0.000469096 | 0.000000000 |
| Arachnida | Araneae | Theridiidae | 0.000247281 | 0.000000000 | 0.000000000 |
| Arachnida | Araneae | no identification | 0.016809618 | 0.037895659 | 0.092347129 |
| Arachnida | Ixodida | no identification | 0.000504433 | 0.000115347 | 0.000000000 |
| Arachnida | Mesostigmata | Parasitidae | 0.000265667 | 0.000209644 | 0.000000000 |
| Arachnida | Mesostigmata | no identification | 0.000738686 | 0.006747551 | 0.000165622 |
| Arachnida | Sarcoptiformes | Achipteriidae | 0.000015600 | 0.000000000 | 0.000000000 |
| Arachnida | Sarcoptiformes | Galumnidae | 0.000000000 | 0.000000000 | 0.000824437 |
| Arachnida | Sarcoptiformes | Malaconothridae | 0.000000000 | 0.000204750 | 0.000000000 |
| Arachnida | Sarcoptiformes | Oppiidae | 0.000000000 | 0.000000000 | 0.000536137 |
| Arachnida | Sarcoptiformes | Scheloribatidae | 0.000036900 | 0.000047000 | 0.009588221 |
| Arachnida | Sarcoptiformes | no identification | 0.002796114 | 0.016544428 | 0.020495344 |
| Arachnida | Scorpiones | no identification | 0.000540312 | 0.000518794 | 0.001295213 |
| Arachnida | Solifugae | no identification | 0.000000000 | 0.000017300 | 0.000000000 |
| Arachnida | Trombidiformes | Bdellidae | 0.000000000 | 0.000000000 | 0.000068300 |
| Arachnida | Trombidiformes | Demodicidae | 0.000056400 | 0.000000000 | 0.000000000 |
| Arachnida | Trombidiformes | Eupodidae | 0.000000000 | 0.000000000 | 0.005636096 |
| Arachnida | Trombidiformes | Scutacaridae | 0.000000000 | 0.000000000 | 0.000082000 |
| Arachnida | Trombidiformes | Tarsonemidae | 0.000000000 | 0.000727173 | 0.000730458 |
| Arachnida | Trombidiformes | no identification | 0.001824164 | 0.001253511 | 0.000058500 |
| Arachnida | no identification | no identification | 0.000214111 | 0.001956087 | 0.000571974 |
| Clitellata | Crassiclitellata | Lumbricidae | 0.727888535 | 0.207607904 | 0.023237235 |
| Clitellata | Enchytraeida | Enchytraeidae | 0.016212639 | 0.004104045 | 0.004435616 |
| Clitellata | Enchytraeida | no identification | 0.000017300 | 0.000000000 | 0.000000000 |
| Clitellata | no identification | no identification | 0.000745458 | 0.000618660 | 0.000872540 |
| Diplopoda | Julida | Julidae | 0.000110972 | 0.000000000 | 0.000000000 |
| Gastropoda | Acochlidiacea | no identification | 0.002515852 | 0.000811096 | 0.000204201 |
| Gastropoda | Architaenioglossa | Viviparidae | 0.000155129 | 0.000000000 | 0.000018700 |
| Gastropoda | Cephalaspidea | no identification | 0.000147269 | 0.000040000 | 0.000114478 |
| Gastropoda | Littorinimorpha | Bithyniidae | 0.016275508 | 0.000161533 | 0.000000000 |
| Gastropoda | Littorinimorpha | Rissoidae | 0.000023200 | 0.000000000 | 0.000004600 |
| Gastropoda | Littorinimorpha | no identification | 0.000000000 | 0.000210438 | 0.000000000 |
| Gastropoda | Neogastropoda | Cystiscidae | 0.000000000 | 0.000022200 | 0.000239042 |
| Gastropoda | Neogastropoda | no identification | 0.000000000 | 0.000000000 | 0.000084100 |
| Gastropoda | Nudibranchia | no identification | 0.001326857 | 0.005517874 | 0.000421057 |
| Gastropoda | Runcinacea | no identification | 0.000000000 | 0.000279442 | 0.000090800 |
| Gastropoda | Stylommatophora | Agriolimacidae | 0.007569622 | 0.005258941 | 0.014642484 |
| Gastropoda | Stylommatophora | Arionidae | 0.000000000 | 0.000000000 | 0.000144991 |
| Gastropoda | Stylommatophora | no identification | 0.001158611 | 0.002081047 | 0.001145809 |
| Gastropoda | Systellommatophora | no identification | 0.000235635 | 0.000034900 | 0.000060900 |
| Gastropoda | no identification | Planorbidae | 0.000362040 | 0.000172862 | 0.001235064 |
| Gastropoda | no identification | Protancylidae | 0.000030200 | 0.000000000 | 0.000195488 |
| Gastropoda | no identification | no identification | 0.007406791 | 0.007100427 | 0.019954672 |
| Insecta | Coleoptera | Carabidae | 0.000114219 | 0.001776768 | 0.007976276 |
| Insecta | Coleoptera | Chrysomelidae | 0.000123407 | 0.000066500 | 0.000119859 |
| Insecta | Coleoptera | Curculionidae | 0.000081600 | 0.000430758 | 0.001332465 |
| Insecta | Coleoptera | Elateridae | 0.000000000 | 0.000086600 | 0.007835627 |
| Insecta | Coleoptera | Histeridae | 0.000000000 | 0.000000000 | 0.001298088 |
| Insecta | Coleoptera | Staphylinidae | 0.000000000 | 0.000524648 | 0.000000000 |
| Insecta | Coleoptera | no identification | 0.006428437 | 0.021472659 | 0.006082484 |
| Insecta | Diptera | Agromyzidae | 0.000000000 | 0.000000000 | 0.000112943 |
| Insecta | Diptera | Bibionidae | 0.002544493 | 0.000028700 | 0.000000000 |
| Insecta | Diptera | Calliphoridae | 0.000000000 | 0.000110394 | 0.000000000 |
| Insecta | Diptera | Cecidomyiidae | 0.000258484 | 0.000110595 | 0.000000000 |
| Insecta | Diptera | Chironomidae | 0.000301025 | 0.005114115 | 0.019873084 |
| Insecta | Diptera | Chloropidae | 0.000000000 | 0.000000000 | 0.000355366 |
| Insecta | Diptera | Culicidae | 0.000000000 | 0.000000000 | 0.000134264 |
| Insecta | Diptera | Drosophilidae | 0.000000000 | 0.000220226 | 0.000000000 |
| Insecta | Diptera | Keroplatidae | 0.000000000 | 0.007365291 | 0.000000000 |
| Insecta | Diptera | Limoniidae | 0.000000000 | 0.000027600 | 0.000000000 |
| Insecta | Diptera | Lonchopteridae | 0.000046900 | 0.000000000 | 0.000000000 |
| Insecta | Diptera | Muscidae | 0.000000000 | 0.000188218 | 0.007160498 |
| Insecta | Diptera | Mycetophilidae | 0.001147269 | 0.004826564 | 0.008585607 |
| Insecta | Diptera | Phoridae | 0.000062300 | 0.000000000 | 0.000000000 |
| Insecta | Diptera | Polleniidae | 0.000136444 | 0.000000000 | 0.000000000 |
| Insecta | Diptera | Psychodidae | 0.000147705 | 0.000000000 | 0.000000000 |
| Insecta | Diptera | Rhagionidae | 0.000129945 | 0.001655973 | 0.000000000 |
| Insecta | Diptera | Scathophagidae | 0.000015200 | 0.006346237 | 0.000000000 |
| Insecta | Diptera | Sciaridae | 0.000077600 | 0.000121212 | 0.000000000 |
| Insecta | Diptera | Syrphidae | 0.001529464 | 0.001586985 | 0.001385276 |
| Insecta | Diptera | Tabanidae | 0.000481622 | 0.000019100 | 0.000109649 |
| Insecta | Diptera | Tipulidae | 0.030784046 | 0.474588632 | 0.584468314 |
| Insecta | Diptera | no identification | 0.008765130 | 0.024216009 | 0.035605936 |
| Insecta | Ephemeroptera | Baetidae | 0.000000000 | 0.000313480 | 0.000000000 |
| Insecta | Hemiptera | Aphididae | 0.000000000 | 0.000477354 | 0.000188239 |
| Insecta | Hemiptera | Cicadellidae | 0.000231286 | 0.000530736 | 0.001048607 |
| Insecta | Hemiptera | no identification | 0.000705357 | 0.000625759 | 0.000502052 |
| Insecta | Hymenoptera | Braconidae | 0.000733506 | 0.000765718 | 0.000064700 |
| Insecta | Hymenoptera | Tenthredinidae | 0.000000000 | 0.000170242 | 0.000000000 |
| Insecta | Hymenoptera | Vespidae | 0.000000000 | 0.000098800 | 0.000000000 |
| Insecta | Hymenoptera | no identification | 0.004665703 | 0.007670289 | 0.005472192 |
| Insecta | Lepidoptera | Crambidae | 0.004040030 | 0.005098184 | 0.007872303 |
| Insecta | Lepidoptera | Geometridae | 0.000000000 | 0.000175925 | 0.000000000 |
| Insecta | Lepidoptera | Gracillariidae | 0.000013400 | 0.000000000 | 0.000331993 |
| Insecta | Lepidoptera | Noctuidae | 0.007936438 | 0.003155627 | 0.000296998 |
| Insecta | Lepidoptera | no identification | 0.032729979 | 0.032973829 | 0.016751123 |
| Insecta | Neuroptera | no identification | 0.000038800 | 0.000863848 | 0.000073400 |
| Insecta | Odonata | no identification | 0.001363263 | 0.000286450 | 0.000330405 |
| Insecta | Siphonaptera | no identification | 0.000323205 | 0.000014100 | 0.000620247 |
| Insecta | Trichoptera | no identification | 0.000000000 | 0.000209127 | 0.000004600 |
| Insecta | no identification | no identification | 0.087044380 | 0.094096605 | 0.082880029 |
| Malacostraca | Amphipoda | no identification | 0.000443526 | 0.000843363 | 0.000215576 |
| Malacostraca | Decapoda | no identification | 0.000000000 | 0.000019100 | 0.000811205 |
| Malacostraca | Isopoda | Philosciidae | 0.000093600 | 0.000000000 | 0.000000000 |

**Table S3.2** The frequency of occurrence of all families within the classes Arachnida, Clitellata, Diplopoda, Gastropoda, Insecta and Malacostraca, in the periods pre-nesting 2022 (n=27 samples), post-nesting 2022 (n=66) and post-nesting 2023 (n=114).

| **Class** | **Order** | **Family** | **Before nesting 2022** | **After nesting 2022** | **After nesting 2023** |
| --- | --- | --- | --- | --- | --- |
| Arachnida | Araneae | Linyphiidae | 7.41 | 0.00 | 2.63 |
| Arachnida | Araneae | Lycosidae | 3.70 | 6.06 | 0.00 |
| Arachnida | Araneae | Theridiidae | 25.93 | 0.00 | 0.00 |
| Arachnida | Araneae | no identification | 100.00 | 95.45 | 93.86 |
| Arachnida | Ixodida | no identification | 11.11 | 3.03 | 0.00 |
| Arachnida | Mesostigmata | Parasitidae | 3.70 | 1.52 | 0.00 |
| Arachnida | Mesostigmata | no identification | 7.41 | 3.03 | 3.51 |
| Arachnida | Sarcoptiformes | Achipteriidae | 3.70 | 0.00 | 0.00 |
| Arachnida | Sarcoptiformes | Galumnidae | 0.00 | 0.00 | 4.39 |
| Arachnida | Sarcoptiformes | Malaconothridae | 0.00 | 1.52 | 0.00 |
| Arachnida | Sarcoptiformes | Oppiidae | 0.00 | 0.00 | 2.63 |
| Arachnida | Sarcoptiformes | Scheloribatidae | 3.70 | 4.55 | 5.26 |
| Arachnida | Sarcoptiformes | no identification | 29.63 | 40.91 | 32.46 |
| Arachnida | Scorpiones | no identification | 33.33 | 6.06 | 21.93 |
| Arachnida | Solifugae | no identification | 0.00 | 1.52 | 0.00 |
| Arachnida | Trombidiformes | Bdellidae | 0.00 | 0.00 | 0.88 |
| Arachnida | Trombidiformes | Demodicidae | 3.70 | 0.00 | 0.00 |
| Arachnida | Trombidiformes | Eupodidae | 0.00 | 0.00 | 1.75 |
| Arachnida | Trombidiformes | Scutacaridae | 0.00 | 0.00 | 0.88 |
| Arachnida | Trombidiformes | Tarsonemidae | 0.00 | 6.06 | 1.75 |
| Arachnida | Trombidiformes | no identification | 14.81 | 3.03 | 0.88 |
| Arachnida | no identification | no identification | 14.81 | 15.15 | 5.26 |
| Clitellata | Crassiclitellata | Lumbricidae | 100.00 | 96.97 | 39.47 |
| Clitellata | Enchytraeida | Enchytraeidae | 40.74 | 28.79 | 21.93 |
| Clitellata | Enchytraeida | no identification | 3.70 | 0.00 | 0.00 |
| Clitellata | no identification | no identification | 33.33 | 6.06 | 5.26 |
| Diplopoda | Julida | Julidae | 3.70 | 0.00 | 0.00 |
| Gastropoda | Acochlidiacea | no identification | 40.74 | 13.64 | 4.39 |
| Gastropoda | Architaenioglossa | Viviparidae | 3.70 | 0.00 | 0.88 |
| Gastropoda | Cephalaspidea | no identification | 7.41 | 1.52 | 1.75 |
| Gastropoda | Littorinimorpha | Bithyniidae | 7.41 | 3.03 | 0.00 |
| Gastropoda | Littorinimorpha | Rissoidae | 3.70 | 0.00 | 0.88 |
| Gastropoda | Littorinimorpha | no identification | 0.00 | 1.52 | 0.00 |
| Gastropoda | Neogastropoda | Cystiscidae | 0.00 | 1.52 | 1.75 |
| Gastropoda | Neogastropoda | no identification | 0.00 | 0.00 | 0.88 |
| Gastropoda | Nudibranchia | no identification | 22.22 | 27.27 | 3.51 |
| Gastropoda | Runcinacea | no identification | 0.00 | 4.55 | 2.63 |
| Gastropoda | Stylommatophora | Agriolimacidae | 44.44 | 15.15 | 14.91 |
| Gastropoda | Stylommatophora | Arionidae | 0.00 | 0.00 | 0.88 |
| Gastropoda | Stylommatophora | no identification | 37.04 | 30.30 | 14.91 |
| Gastropoda | Systellommatophora | no identification | 14.81 | 1.52 | 0.88 |
| Gastropoda | no identification | Planorbidae | 7.41 | 6.06 | 15.79 |
| Gastropoda | no identification | Protancylidae | 3.70 | 0.00 | 1.75 |
| Gastropoda | no identification | no identification | 88.89 | 66.67 | 57.89 |
| Insecta | Coleoptera | Carabidae | 11.11 | 18.18 | 11.40 |
| Insecta | Coleoptera | Chrysomelidae | 11.11 | 1.52 | 1.75 |
| Insecta | Coleoptera | Curculionidae | 14.81 | 10.61 | 8.77 |
| Insecta | Coleoptera | Elateridae | 0.00 | 1.52 | 8.77 |
| Insecta | Coleoptera | Histeridae | 0.00 | 0.00 | 0.88 |
| Insecta | Coleoptera | Staphylinidae | 0.00 | 7.58 | 0.00 |
| Insecta | Coleoptera | no identification | 77.78 | 69.70 | 43.86 |
| Insecta | Diptera | Agromyzidae | 0.00 | 0.00 | 0.88 |
| Insecta | Diptera | Bibionidae | 33.33 | 1.52 | 0.00 |
| Insecta | Diptera | Calliphoridae | 0.00 | 1.52 | 0.00 |
| Insecta | Diptera | Cecidomyiidae | 3.70 | 1.52 | 0.00 |
| Insecta | Diptera | Chironomidae | 22.22 | 16.67 | 13.16 |
| Insecta | Diptera | Chloropidae | 0.00 | 0.00 | 0.88 |
| Insecta | Diptera | Culicidae | 0.00 | 0.00 | 0.88 |
| Insecta | Diptera | Drosophilidae | 0.00 | 1.52 | 0.00 |
| Insecta | Diptera | Keroplatidae | 0.00 | 4.55 | 0.00 |
| Insecta | Diptera | Limoniidae | 0.00 | 1.52 | 0.00 |
| Insecta | Diptera | Lonchopteridae | 3.70 | 0.00 | 0.00 |
| Insecta | Diptera | Muscidae | 0.00 | 1.52 | 8.77 |
| Insecta | Diptera | Mycetophilidae | 29.63 | 36.36 | 40.35 |
| Insecta | Diptera | Phoridae | 3.70 | 0.00 | 0.00 |
| Insecta | Diptera | Polleniidae | 3.70 | 0.00 | 0.00 |
| Insecta | Diptera | Psychodidae | 3.70 | 0.00 | 0.00 |
| Insecta | Diptera | Rhagionidae | 7.41 | 6.06 | 0.00 |
| Insecta | Diptera | Scathophagidae | 3.70 | 6.06 | 0.00 |
| Insecta | Diptera | Sciaridae | 3.70 | 1.52 | 0.00 |
| Insecta | Diptera | Syrphidae | 37.04 | 21.21 | 14.91 |
| Insecta | Diptera | Tabanidae | 14.81 | 1.52 | 0.88 |
| Insecta | Diptera | Tipulidae | 92.59 | 100.00 | 97.37 |
| Insecta | Diptera | no identification | 96.30 | 80.30 | 92.11 |
| Insecta | Ephemeroptera | Baetidae | 0.00 | 1.52 | 0.00 |
| Insecta | Hemiptera | Aphididae | 0.00 | 3.03 | 0.88 |
| Insecta | Hemiptera | Cicadellidae | 25.93 | 12.12 | 7.89 |
| Insecta | Hemiptera | no identification | 44.44 | 9.09 | 8.77 |
| Insecta | Hymenoptera | Braconidae | 18.52 | 13.64 | 2.63 |
| Insecta | Hymenoptera | Tenthredinidae | 0.00 | 1.52 | 0.00 |
| Insecta | Hymenoptera | Vespidae | 0.00 | 4.55 | 0.00 |
| Insecta | Hymenoptera | no identification | 77.78 | 53.03 | 47.37 |
| Insecta | Lepidoptera | Crambidae | 11.11 | 9.09 | 8.77 |
| Insecta | Lepidoptera | Geometridae | 0.00 | 1.52 | 0.00 |
| Insecta | Lepidoptera | Gracillariidae | 3.70 | 0.00 | 1.75 |
| Insecta | Lepidoptera | Noctuidae | 11.11 | 10.61 | 1.75 |
| Insecta | Lepidoptera | no identification | 92.59 | 86.36 | 63.16 |
| Insecta | Neuroptera | no identification | 3.70 | 7.58 | 0.88 |
| Insecta | Odonata | no identification | 14.81 | 7.58 | 1.75 |
| Insecta | Siphonaptera | no identification | 14.81 | 1.52 | 10.53 |
| Insecta | Trichoptera | no identification | 0.00 | 6.06 | 0.88 |
| Insecta | no identification | no identification | 100.00 | 98.48 | 97.37 |
| Malacostraca | Amphipoda | no identification | 25.93 | 6.06 | 4.39 |
| Malacostraca | Decapoda | no identification | 0.00 | 1.52 | 9.65 |
| Malacostraca | Isopoda | Philosciidae | 3.70 | 0.00 | 0.00 |

**Table S3.3.** Ten families detected in godwit faeces with the highest deviance values, contributing most to the differences between the diet composition of flock-foraging godwits pre- and post-nesting in 2022. Deviance and p-values were retrieved from a multivariate generalized linear model with the diet composition matrix as response variable and period (categorical: pre- and post-nesting) as predictor value, using the *anova.manyglm* function in R with the p.uni=”adjusted” argument from the package *mvabund* (Wang et al., 2012).

| Class | Order | Family | Deviance | p-value |
| --- | --- | --- | --- | --- |
| Insecta | Diptera | Tipulidae | 15.82 | 0.001 |
| Clitellata | Crassiclitellata | Lumbricidae | 12.84 | 0.001 |
| Gastropoda | Littorinimorpha | Bithyniidae | 0.99 | 0.001 |
| Arachnida | Sarcoptiformes | Unidentified Sarcoptiformes | 0.37 | 0.001 |
| Insecta | Diptera | Keroplatidae | 0.33 | 0.001 |
| Clitellata | Enchytraeida | Enchytraeidae | 0.33 | 0.001 |
| Insecta | Coleoptera | Unidentified Coleoptera | 0.30 | 0.001 |
| Arachnida | Araneae | Unidentified Araneae | 0.30 | 0.001 |
| Insecta | Diptera | Scathophagidae | 0.28 | 0.001 |
| Insecta | Diptera | Unidentified Diptera | 0.27 | 0.001 |

**Table S3.4.** Power analysis comparing the diet composition between the pre-nesting and post-nesting period 2022. The power analysis consisted in randomly resampling the original data set for five iterations, with reposition, to keep only eight locations out of 19 from the post-nesting period each time. Subsequently, the diet composition between the two periods was compared using multivariate Generalized Linear Models.

| Model | Deviance | p-value |
| --- | --- | --- |
| #1 Period | 39.24 | **0.001** |
| #2 Period | 23.38 | **0.001** |
| #3 Period | 24.84 | **0.001** |
| #4 Period | 28.75 | **0.001** |
| #5 Period | 24.38 | **0.001** |

**Table S3.5.** Power analysis comparing the relative read abundance (RRA) of Lumbricidae and Tipulidae between the pre-nesting and post-nesting period 2022. The power analysis consisted in randomly resampling the original data set for five iterations, with reposition, to keep only eight locations out of 19 from the post-nesting period each time. Subsequently, we compared the RRA of Lumbricidae and Tipulidae between the two periods using univariate Generalized Linear Mixed Models. For each model, the post-nesting period was included as the reference.

|  | Estimate | SE | z-value | p-value |
| --- | --- | --- | --- | --- |
| *Lumbricidae* |  |  |  |  |
| #1 |  |  |  |  |
| Intercept | -1.28 | 0.52 | -2.47 | **0.013** |
| Period: Pre-nesting | 2.26 | 0.67 | 3.36 | **<0.001** |
| #2 |  |  |  |  |
| Intercept | -1.66 | 0.50 | -3.33 | **<0.001** |
| Period: Pre-nesting | 2.64 | 0.66 | 4.00 | **<0.001** |
| #3 |  |  |  |  |
| Intercept | -0.95 | 0.41 | -2.30 | **0.021** |
| Period: Pre-nesting | 1.94 | 0.60 | 3.23 | **0.001** |
| #4 |  |  |  |  |
| Intercept | -1.12 | 0.46 | -2.41 | **0.016** |
| Period: Pre-nesting | 2.10 | 0.63 | 3.32 | **<0.001** |
| #5 |  |  |  |  |
| Intercept | -1.23 | 0.45 | -2.73 | **0.006** |
| Period: Pre-nesting | 2.22 | 0.63 | 3.54 | **<0.001** |
|  |  |  |  |  |
| *Tipulidae* |  |  |  |  |
| #1 |  |  |  |  |
| Intercept | -0.11 | 0.38 | -0.29 | 0.774 |
| Period: Pre-nesting | -3.34 | 1.18 | -2.84 | **0.004** |
| #2 |  |  |  |  |
| Intercept | -0.10 | 0.37 | -0.27 | 0.790 |
| Period: Pre-nesting | -3.35 | 1.17 | -2.86 | **0.004** |
| #3 |  |  |  |  |
| Intercept | 0.01 | 0.38 | 0.04 | 0.969 |
| Period: Pre-nesting | -3.46 | 1.18 | -2.94 | **0.003** |
| #4 |  |  |  |  |
| Intercept | 0.178 | 0.38 | 0.47 | 0.639 |
| Period: Pre-nesting | -3.63 | 1.18 | -3.08 | **0.002** |
| #5 |  |  |  |  |
| Intercept | -0.34 | 0.38 | -0.89 | 0.371 |
| Period: Pre-nesting | -3.11 | 1.18 | -2.64 | **0.008** |

**Table S3.6.** Post-hoc analysis of the relationships between soil moisture content and soil resistance with the Ivlev Electivity Index values of Lumbricidae and Tipulidae pre- and post-nesting 2022.

|  | Estimate | SE | z-ratio | p-value |
| --- | --- | --- | --- | --- |
| *E Lumbricidae ~ soil moisture content* |  |  |  |  |
| Pre-nesting | 0.000 | 0.003 | 0.306 | 0.760 |
| Post-nesting | 0.015 | 0.005 | 2.992 | **<0.003** |
| *E Lumbricidae ~ soil resistance* |  |  |  |  |
| Pre-nesting | 0.001 | 0.001 | 0.563 | 0.573 |
| Post-nesting | -0.001 | 0.001 | -2.343 | **0.019** |
| *E Lumbricidae ~ vegetation height* |  |  |  |  |
| Pre-nesting | 0.010 | 0.032 | 0.315 | 0.753 |
| Post-nesting | -0.013 | 0.031 | -0.433 | 0.665 |
|  |  |  |  |  |
| *E tipulidae ~ soil moisture content* |  |  |  |  |
| Pre-nesting | -0.013 | 0.013 | -1.059 | 0.290 |
| Post-nesting | 0.000 | 0.003 | -0.308 | 0.758 |
| *E tipulidae ~ soil resistance* |  |  |  |  |
| Pre-nesting | 0.001 | 0.006 | 0.176 | 0.860 |
| Post-nesting | -0.000 | 0.000 | -0.514 | 0.607 |
| *E tipulidae ~ vegetation height* |  |  |  |  |
| Pre-nesting | 0.165 | 0.157 | 1.048 | 0.295 |
| Post-nesting | 0.032 | 0.015 | 2.207 | **0.027** |
