# Supplementary material for: Soil Moisture and Prey Life Cycles Promote Seasonal Dietary Shift in a Grassland‐Breeding Migratory Shorebird
Source: Ecol Evol. 2026 Oct 11;16(10):e74495. doi: 10.1002/ece3.74495 (PMC13655214; doi:10.1002/ece3.74495)
Supplement: Supplementary file 3 [file ECE3-16-e74495-s003.docx]

Appendix S1

**From:** Soil moisture and prey life cycles promote seasonal dietary shift in a grassland-breeding migratory shorebird species

*Taxonomic assignment of ASVs*

ASVs were taxonomically assigned using both the core_nt database of GenBank (using a local copy created on 28 January 2025 (Benson et al., 2009)) and Barcode of Life Data System (BOLD; using a local copy created on 11 March 2024 (Ratnasingham and Hebert, 2007)) as reference databases. The taxonomy of ASVs with different assignments provided by GenBank and BOLD was resolved according to the following set of rules. (1) ASVs that were assigned as fungi and bacteria in GB but not in BOLD were removed. (2) For ASVs identified in GB at the phylum level with lower taxonomic ranks remaining unclassified, and identified in BOLD to a lower taxonomic level than phylum, the classification of GenBank was used. (3) ASVs that were not taxonomically assigned by BOLD were set to the assignment of GB. (4) For ASVs that were not assigned to a lower taxonomic level than Animalia, Arthropoda or Insecta in BOLD, the classification of GB was used. (5) When ASVs assigned in BOLD as Arthropoda, Annelida, Mollusca, Chordata (phyla of interest for this study) but in GB **not** as Metazoa, the taxonomy assigned by GB was adhered to. (6) When ASVs were assigned by GB as *Homo sapiens*, this taxonomy was used. (7) For ASVS that share higher-level taxonomic assignments between GB and BOLD, but are assigned to a lower taxonomic level in GB (i.e. *Lumbricus rubellus* in GB and *Lumbricus* in BOLD), the taxonomy of GB was used. (8) ASVs assigned as ‘top hits’ in GB but not in BOLD, were assigned according to GB. (9) ASVs that were assigned ‘top hits’ in both databases but differ at the species, genus, family, order or class level for the phyla Annelida, Mollusca and Chordata were checked manually. (10) For ASVs assigned as Arthropoda, all assignments that differed at the order or class level between the two databases were checked manually, but for the remaining assignments that differed at the species, genus or family level, the taxonomy of the lowest common ancestor was used. (11) All ASVs that were no ‘top hits’ (lca), were set to the taxonomy of the lowest common ancestor between BOLD and GenBank.

Following this, ASVS with multiple taxonomic assignments were summarized. We selected all ASVs in the kingdom Animalia that occurred with more than 10 reads in our dataset. Of those, 56 ASVS with the highest read abundance in our dataset were checked manually. For the remaining ASVs, we chose the assignment with highest number of top hits in GB. If this still resulted in multiple ASVS assignments, we chose the assignment with the highest identity. Lastly, taxonomic assignments were checked against the Dutch Species Register, and species not occurring in the Netherlands were corrected accordingly.
